# Localized Netrins Act as Positional Cues to Control Layer-Specific Targeting of Photoreceptor Axons in *Drosophila*

**DOI:** 10.1016/j.neuron.2012.04.037

**Published:** 2012-07-12

**Authors:** Katarina Timofeev, Willy Joly, Dafni Hadjieconomou, Iris Salecker

**Affiliations:** 1 Division of Molecular Neurobiology, MRC National Institute for Medical Research, London NW7 1AA, UK

## Abstract

A shared feature of many neural circuits is their organization into synaptic layers. However, the mechanisms that direct neurites to distinct layers remain poorly understood. We identified a central role for Netrins and their receptor Frazzled in mediating layer-specific axon targeting in the *Drosophila* visual system. Frazzled is expressed and cell autonomously required in R8 photoreceptors for directing their axons to the medulla-neuropil layer M3. Netrin-B is specifically localized in this layer owing to axonal release by lamina neurons L3 and capture by target neuron-associated Frazzled. Ligand expression in L3 is sufficient to rescue R8 axon-targeting defects of Netrin mutants. R8 axons target normally despite replacement of diffusible Netrin-B by membrane-tethered ligands. Finally, Netrin localization is instructive because expression in ectopic layers can retarget R8 axons. We propose that provision of localized chemoattractants by intermediate target neurons represents a highly precise strategy to direct axons to a positionally defined layer.

## Highlights

► Netrins selectively target Frazzled-expressing R8 photoreceptor axons to layer M3 ► Precise positional information is generated by Netrin localization in a single layer ► Local axonal release and receptor-mediated capture restrict ligand distribution ► Layer-specific targeting relies on ligand release by intermediate target neurons

## Teaser

Many neural circuits share a remarkable organization into synaptic layers. Timofeev et al. find that in the *Drosophila* visual system, Netrins act as short-range chemoattractants to target photoreceptor axons, specifically expressing the Frazzled/DCC/Unc-40 receptor, to a single positionally defined layer.

## Introduction

The formation of specific synaptic connections between distinct sets of afferent axons and partner neurons during development is pivotal for normal brain function in vertebrates and invertebrates. Larger neural circuits are frequently subdivided into reiterated columnar and layered local circuits. This anatomical organization particularly applies to the visual system, where columnar modules form a topographic map to represent visual space, while layered units are instrumental for parallel integration of visual information such as motion or spectral sensitivity (Sanes and Zipursky, 2010). Moreover, during development this architecture helps to spatially group potential synaptic partners and therefore restrict the number of possible contacts in an otherwise large connectivity matrix (Huberman et al., 2010). However, despite their importance for function and development, our understanding as to how the formation of layer-specific connections is controlled at the molecular and cellular level is still limited.

**Figure 1:**
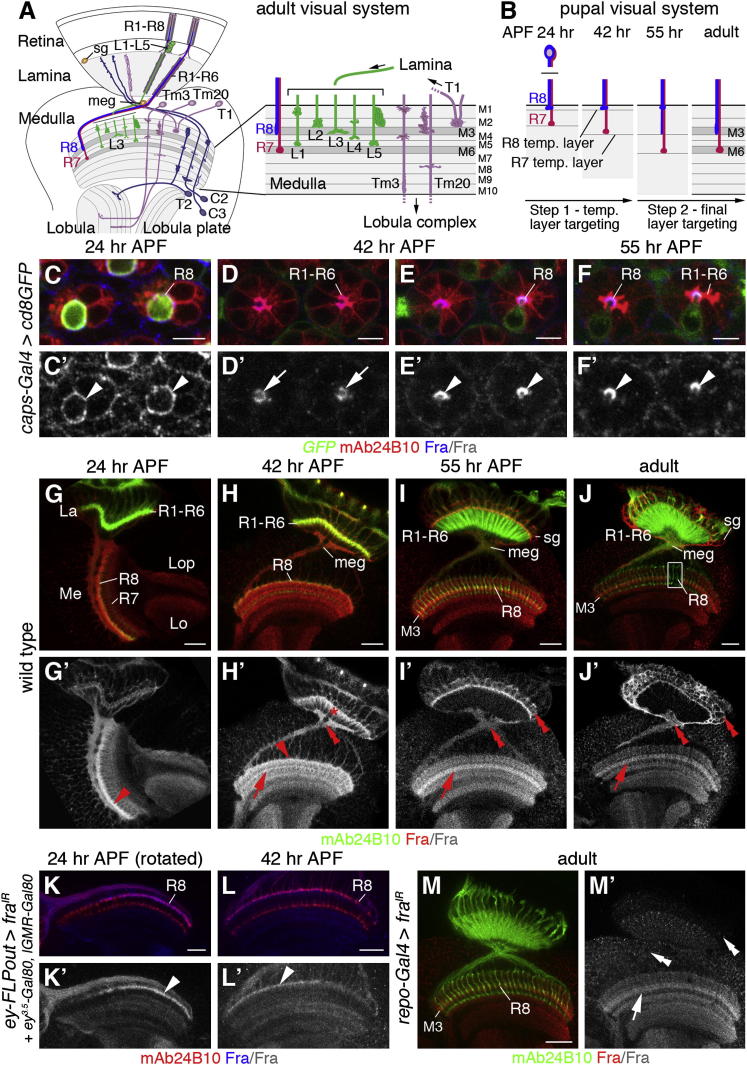
Fra Is Expressed in R Cells and the Optic Lobe (A) Schematic of the *Drosophila* adult visual system highlighting the organization of the medulla neuropil into ten layers (M1–M10), and the neuron subtypes relevant for this study. sg, satellite glia; meg, medulla glia. (B) Schematic illustrates of R8 and R7 axon-targeting steps to temporary and final M3 and M6 layers during metamorphosis. (C–F′) In the retina, Fra (blue) is expressed along the cell body membranes of R8 cells at 24 hr (arrowheads in C′) and in rhabdomeres at 42 and 55 hr APF (arrowheads in E′ and F′). Fra is expressed in R1–R6 rhabdomeres at 42 hr (arrows in D′). R8 cells are labeled with *caps-Gal4* and *UAS-cd8GFP* (green). (G–J′) In the medulla (Me), Fra (red) is enriched in the temporary layer of R8 axons at 24 and 42 hr (arrowheads in G′ and H′) and in the emerging and final M3 layer at 42 and 55 hr and in adults (arrows in H′, I′, and J′). In the lamina (La), Fra is enriched in R1–R6 axons at 42 hr (asterisk in H′). sg and meg glial subtypes are Fra positive (double arrowheads in H′, I′, and J′). Lo, lobula; Lop, lobula plate. (K–L′) Target area-specific knockdown reveals Fra expression in R8 axons at 24 and 42 hr (arrowheads in K′ and L′). (M and M′) Knockdown of *fra* using *repo-Gal4* reduces expression in glia (double arrowheads in M′), but not in the M3 layer (arrow in M′), and does not affect R cell axon targeting. R cells are labeled with mAb24B10 (red in C–F′ and K–L′; green in G–J′, M, and M′). Scale bars, 5 μm (C–F) and 20 μm (G–M). See also Figure S1.

The *Drosophila* visual system is characterized by a remarkable organization into parallel synaptic layers (Hadjieconomou et al., 2011b; Sanes and Zipursky, 2010). The retina consists of approximately 800 ommatidia, each containing eight photoreceptor subtypes (R cells, R1–R8). Their axons extend into the optic lobe, where they connect with target neurons in two ganglia: R1–R6 axons project into the lamina, while R8 and R7 axons terminate in the medulla (Figure 1A). Neurites in the medulla are organized into ten synaptic layers (M1–M10) with R8 and R7 axons terminating in the layers M3 and M6, respectively. Similarly, target neurons including lamina neurons L1–L5, medulla neurons, and ascending higher-order neurons arborize within one or more of these ten layers in defined patterns (Fischbach and Dittrich, 1989; Morante and Desplan, 2008). Medulla layers assemble stepwise during metamorphosis following interdependent cell-type-specific programs. R8 and R7 axons are initially positioned in temporary layers and then proceed to their final layers during midpupal development (Ting et al., 2005) (Figure 1B). Previous studies implicated the nonclassical Cadherin Flamingo (Fmi) (Hakeda-Suzuki et al., 2011; Senti et al., 2003), the transmembrane protein Golden goal (Gogo) (Hakeda-Suzuki et al., 2011; Mann et al., 2012; Tomasi et al., 2008), and the leucine-rich repeat protein Capricious (Caps) in R8 axon targeting (Shinza-Kameda et al., 2006). While these studies could explain how layer-specific connections of afferent and target neurons are assembled through control of adhesiveness, the mechanisms that precisely position their neurites within one emerging layer remained unclear.

Netrins are secreted chemotropic guidance molecules related to Laminin (Harris et al., 1996; Ishii et al., 1992; Kennedy et al., 1994; Lai Wing Sun et al., 2011; Mitchell et al., 1996; Serafini et al., 1994, 1996). They elicit an attractive growth cone response by engaging the receptor Frazzled (Fra) (Kolodziej et al., 1996), the *Drosophila* homolog of Unc-40 in *C. elegans* (Chan et al., 1996), and Deleted in Colorectal Cancer (DCC) in vertebrates (Höpker et al., 1999; Keino-Masu et al., 1996), and a repellent response by activation of the Unc-5 receptor (Hong et al., 1999; Keleman and Dickson, 2001; Leonardo et al., 1997; Leung-Hagesteijn et al., 1992). Netrins and Fra/DCC/Unc-40 are well known for their phylogenetically conserved role in orchestrating axon guidance and dendritic growth, as well as glial cell migration relative to the central nervous system (CNS) midline (Brierley et al., 2009; Dickson and Zou, 2010; Evans and Bashaw, 2010; Harris et al., 1996; Hedgecock et al., 1990; Ishii et al., 1992; Kennedy et al., 1994; Lai Wing Sun et al., 2011; Mauss et al., 2009; Mitchell et al., 1996; Serafini et al., 1994, 1996; von Hilchen et al., 2010). Furthermore, their functions extend to the regulation of axonal pathfinding into the optic nerve head (Deiner et al., 1997), topographic sorting of thalamocortical axon projections in the vertebrate brain (Powell et al., 2008), synaptogenesis by influencing axon branch extensions in the CNS (Manitt et al., 2009) and on muscles (Labrador et al., 2005; Winberg et al., 1998), and myelin-like membrane sheet formation of glia (Jarjour et al., 2003). In the *Drosophila* third-instar larval visual system, previous studies have shown that *fra* is nonautononomously required for R cell axon bundle spacing (Gong et al., 1999). However, as to whether this guidance system could regulate layer-specific connectivity was not known.

Here, we show that the Netrin-Fra/DCC/Unc-40 guidance system plays a pivotal role in controlling layer-specific targeting in the *Drosophila* visual system. During metamorphosis, R8 axons express Fra, while Netrins are restricted to a single medulla-neuropil layer, the R8 axon-recipient layer M3. Genetic perturbation of Netrins and Fra results in a failure of R8 axons to extend to their correct target layer. Netrins are locally released by the axon terminals of lamina neurons L3 and, instead of forming a gradient, are captured by Fra-expressing target neuron branches in layer M3. Localized Netrins act at short range and are instructive for layer-specific targeting. Our findings provide evidence that localized chemoattractant guidance molecules released not by the synaptic partners but by intermediate target neurons can coordinate layer-specific targeting of axons by providing distinct positional information.

## Results

### Fra Is Expressed in R Cells and Target Neurons in the Optic Lobe

To gain insights into the role of the Fra guidance receptor in adult visual circuit assembly, we examined its expression in the retina and optic lobe. In the retina (Figures 1C–1F′), colabeling with *capricious-Gal4* (*caps-Gal4*) (Shinza-Kameda et al., 2006) driving membrane-bound green fluorescent protein (GFP) expression revealed that at 24 hr after puparium formation (APF), Fra protein is expressed in R8 cells along their cell bodies, and at 42 and 55 hr in their rhabdomeres, the membrane-rich organelles required for phototransduction in adults. Fra was also transiently detected in rhabdomeres of R1–R6 cells at 42 hr. In the optic lobe (Figures 1G–1J′), Fra protein initially accumulates at the distal medulla neuropil border, where R8 axons temporarily pause before proceeding to their final layer M3 during the second half of pupal development. Specific knockdown of *fra* in the target area by expressing a UAS RNA interference (RNAi) transgene (*UAS-fra^IR^*) using the FLPout approach (Ito et al., 1997) in conjunction with the transgenes *ey-FLP* (Newsome et al., 2000), *ey^3.5^-Gal80* (Chotard et al., 2005), and *longGMR-Gal80* (*lGMR*, kindly provided by C. Desplan) (Wernet et al., 2003) indicated that this expression can be attributed to R8 growth cones (Figures 1K–1L′). At 42 and 55 hr, Fra protein is enriched in the emerging and final M3 layer (Figures 1H–1I′). Expression persists at lower levels in adults (Figures 1J and 1J′). Moreover, Fra is strongly expressed in R1–R6 axons in the lamina at 42 hr, when their growth cones leave their original bundle and extend stereotypic projections to adjacent columns (Figures 1H and 1H′). Additional expression was detected in glial cell subtypes in the lamina and medulla. However, within the medulla neuropil, Fra expression is associated with neurons because glial-specific knockdown using *reversed polarity (repo)-Gal4* (Sepp and Auld, 2003) did not alter the expression pattern (Figures 1M and 1M′). Knockdown of *fra* specifically in the eye using the FLPout approach in conjunction with the *ey^3.5^-FLP* transgene (Bazigou et al., 2007) further confirmed that Fra protein is associated with target neuron processes (see Figure S1 available online). Thus, Fra is expressed by R8 axons and in neurites of target neuron subtypes extending into the M3 layer.

### *fra* Is Cell Autonomously Required for Targeting of R8 Axons to the M3 Layer

**Figure 2:**
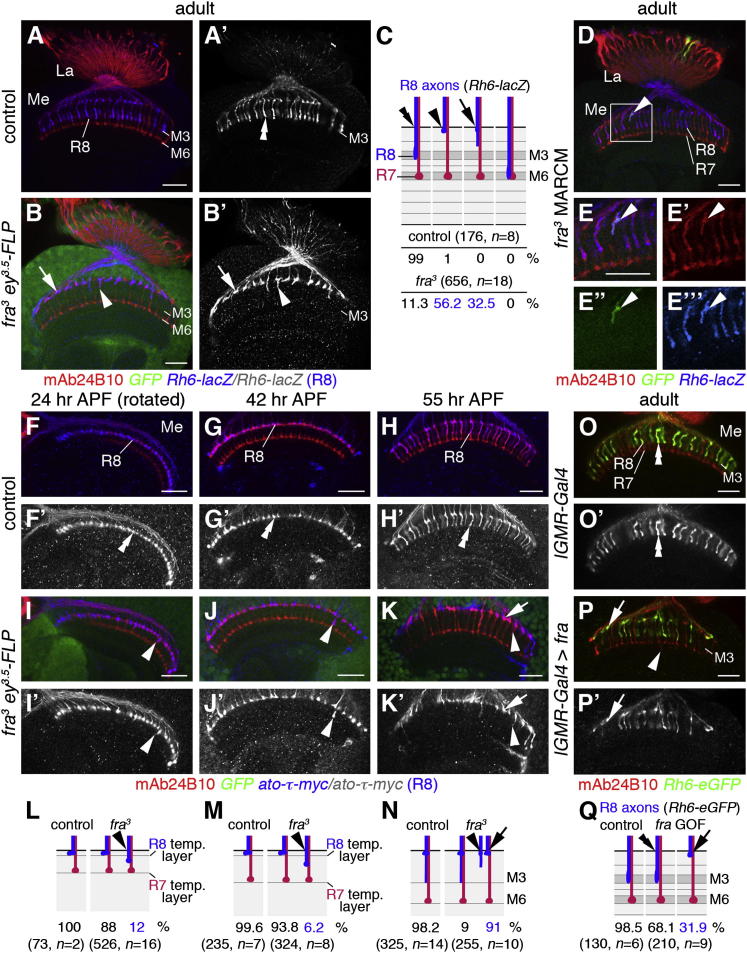
*fra* Is Required in R Cells to Regulate R8 Axon Targeting to the M3 Layer (A and A′) In controls, *Rh6-lacZ*-positive R8 axons (blue) target to the M3 layer in the medulla (Me) (double arrowhead in A′). La, lamina. (B and B′) In *ey^3.5^-FLP* mosaic animals lacking *fra* in R cells, many R8 axons stall at the medulla neuropil border (arrows) or terminate incorrectly in the M1/M2 layers (arrowheads). (C) Quantification of phenotypes is shown. (D–E″′) A *fra* mutant, GFP-positive R8 axon generated by MARCM terminates incorrectly in M1/M2 (arrowheads). The area outlined in (D) is shown at higher magnification in (E)–(E″′). (F–G′) At 24 and 42 hr APF, all R8 axons expressing *ato-τ-myc* (blue) terminate in their temporary layer at the medulla neuropil border in controls (double arrowheads in F′ and G′). (H and H′) At 55 hr, all R8 axons proceed to the M3 layer in the medulla neuropil in controls (double arrowheads). (I–J′) In animals lacking *fra* in R cells, the majority of R8 axons pause at the medulla neuropil border, whereas a small percentage prematurely projects deeper (arrowheads). (K and K′) In *fra ey^3.5^-FLP* mosaics, many R8 axons fail to enter the medulla neuropil (arrow) or terminate prematurely in the M1/M2 layers (arrowhead). (L–N) Quantification of phenotypes is presented. (O–P′) Overexpression of *fra* in R cells causes stalling of *Rh6-eGFP*-positive R8 axons (green) at the medulla neuropil border (arrows in P and P′), when compared to controls (double arrowheads in O and O′). R7 axons (red) are not retargeted to the M3 layer but occasionally show small extensions into deeper layers (arrowheads in P and P′). (Q) Quantification of phenotypes is shown. R cells are labeled with mAb24B10 (red). Scale bars, 20 μm. See also Figures S2 and S3.

To assess the function of *fra* in controlling R cell axon targeting, we used the *ey^3.5^-FLP* approach to render the majority of R cells homozygous mutant, while leaving wild-type activity in the target area (Bazigou et al., 2007; Newsome et al., 2000). We focused on the well-characterized loss-of-function *fra^3^* allele (Kolodziej et al., 1996), as eye development was not affected (Figure S2). Within the optic lobe of these adult mosaic animals, 88.7% of *fra^3^* mutant R8 axons, identified with the marker *Rh6-lacZ* (656 axons, n = 18), exhibited strong projection defects: 56.2% stalled at the medulla neuropil border, while 32.5% terminated prematurely at the more distal layers M1 and M2 (Figures 2A–2C). In contrast, ganglion-specific targeting of R1–R6 axons to the lamina and layer-specific targeting of R7 axons to the M6 layer appeared unaffected (Figure S3). Single *Rh6-lacZ-*positive *fra^3^* homozygous mutant R8 axons generated by mosaic analysis with a repressible cell marker (MARCM) (Lee and Luo, 1999) showed fully penetrant phenotypes: they either stalled at the distal medulla neuropil border (5 of 17 clones) or terminated in the M1/M2 layers (12 of 17 clones), while neighboring heterozygous R8 axons terminated correctly in the M3 layer (Figures 2D–2E″′). Hence, *fra* is required cell autonomously in R8 neurons for targeting to the correct layer.

To assess at which targeting step these defects occur during metamorphosis, R8 axons were labeled with the early marker *ato-τ-myc* (Bazigou et al., 2007; Senti et al., 2003) (Figures 2F–2N). From 24 hr APF onward, all wild-type R8 axons are located in the temporary layer at the distal medulla neuropil border, where they pause for approximately 30 hr before projecting to the emerging recipient layer M3 at around 55 hr APF (Ting et al., 2005). In *ey^3.5^-FLP* mosaics, a small proportion of *fra* mutant R8 axons at 24 hr (12%, 526 axons, n = 16) and 42 hr (6.2%, 324 axons, n = 8) proceeded prematurely into the neuropil located between R8 and R7 growth cones. At 55 hr, the majority of mutant R8 axons stalled at the medulla neuropil border (91%, 255 axons, n = 10). This indicates that *fra* is mainly required during the second targeting step to the final layer. Histological analysis and immunolabeling with available markers showed that the observed phenotypes are not the consequence of general eye development errors, R8 cell fate-specification defects, abnormal proliferation and differentiation of target neurons and glia, or earlier R cell-projection defects during the third-instar larval stage (Figure S2).

To test whether *fra* is also sufficient, we expressed this receptor in all R cells using *lGMR-Gal4* as driver. This prolonged ectopic expression did not redirect R7 axons to the M3 layer, while many R8 axons (31.9% of 210 *Rh6-lacZ*-positive axons, n = 9) remained in the temporary layer (Figures 2O–2Q). Thus, expression of Fra in R cells is not sufficient to alter target layer specificity.

### Expression of Netrins in the Optic Lobe

**Figure 3:**
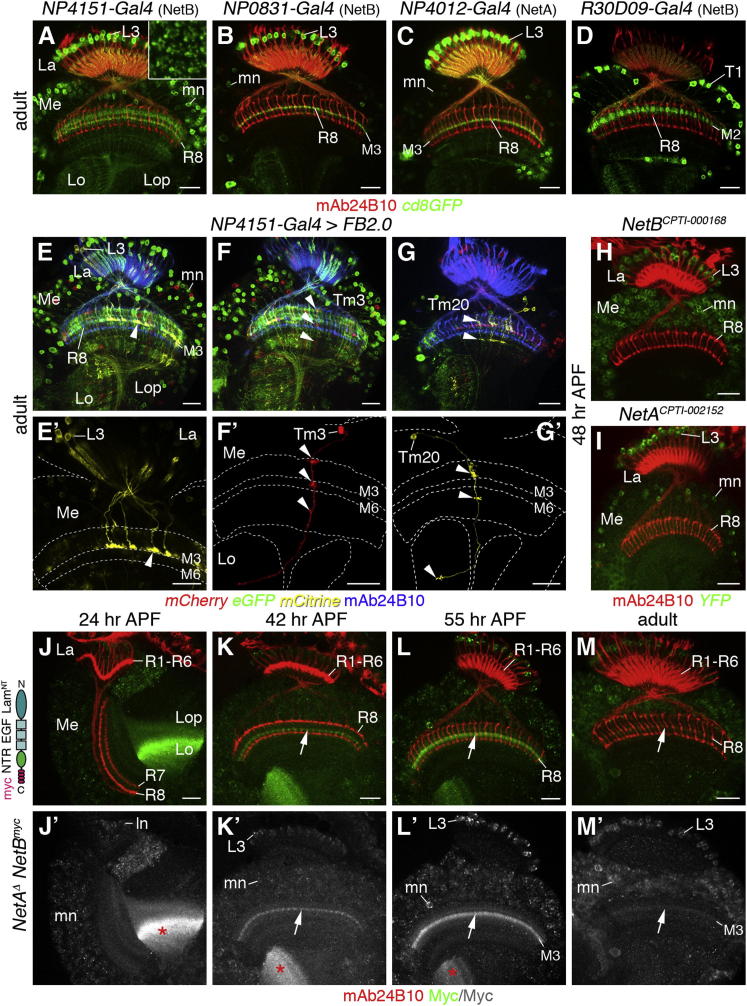
NetB Expression in the Optic Lobe (A–C) *NP4151*-, *NP0831-*, and *NP4012-Gal4* insertions adjacent to *NetB* and *NetA* loci drive GFP expression (green) in lamina (La) neurons L3, which extend axon terminals into layer M3, and in medulla (Me) neuron subtypes (mn). Inset in (A) presents cross sections through lamina cartridges showing the asymmetric dendritic arbors of lamina neurons L3. Lo, lobula; Lop, lobula plate. (D) *R30D09-Gal4* identifies T1 neurons among NetB-expressing medulla neurons. (E–G′) Mapping of Netrin-producing neuron subtypes using the *FB2.0* approach and *NP4151-Gal4* identifies lamina neurons L3 (yellow, arrowheads in E and E′), and the transmedullary neurons Tm3 (n = 8, red in F and F′) and Tm20 (n = 11, yellow in G and G′) based on their characteristic arborizations within the medulla and lobula (arrowheads). Reconstructions of neurons expressing mCitrine or mCherry are shown in (E′), (F′), and (G′). (H and I) Protein trap insertions into *NetB* (H) and *NetA* (I) show GFP expression in lamina neurons L3 and some medulla neurons at 48 hr APF. (J–M′) Schematic of NetB^myc^ shows the laminin N-terminal domain (Lam^NT^), EGF repeats, Netrin-like domain (NTR), and myc tag. Animals with *NetB^myc^* (green) knocked into its endogenous locus in *NetA* mutants show NetB protein in lamina neuron (ln) and medulla neuron (mn) cell bodies and an enrichment in the emerging M3 layer at 42 and 55 hr (arrows in K–L′) and in the lobula neuropil (asterisks in J′, K′, and L′). Expression decreases in adults (arrows in M and M′). R cells are labeled with mAb24B10 (red in A–D and H–M; blue in E, F, and G). Scale bars, 20 μm. See also Figure S4.

Focusing next on the activating ligands of Fra, Netrin-A and Netrin-B (NetA, NetB) (Harris et al., 1996; Mitchell et al., 1996), we set out to identify the neurons that act as their potential sources in the adult and pupal optic lobe. Examining enhancer trap *Gal4* P element insertions (Hayashi et al., 2002) into or close to the *NetA* and *NetB* genomic loci, we observed reporter gene expression in lamina neurons L3, which extend axonal arbors into the same layer as R8 axons, as well as in medulla neuron subtypes (Figures 3A–3C). NetB-positive neuron subtypes were mapped using *R30D09-Gal4*, a driver under the control of a defined *NetB* enhancer fragment (Pfeiffer et al., 2008), as well as *NP4151-Gal4* in conjunction with the genetic multicolor cell-labeling approach *Flybow* (*FB*) *2.0* (Hadjieconomou et al., 2011a). Neuron subtypes were identified based on their previously described branching patterns (Fischbach and Dittrich, 1989). This showed that the NetB-expressing neuron population, in addition to lamina neurons L3, comprises ascending T1 neurons, which connect the medulla and lamina (Figure 3D), the transmedullary neuron subtypes Tm3, Tm20, Tm2, Tm5, Tm13, Tm14, and Tm25, which extend from the medulla into the lobula, and T2 neurons, which connect the lobula and lobula plate with the medulla (Figures 3E–3G′; data not shown). Comparison of YFP-trap insertions (Ryder et al., 2009) into *NetA* and *NetB* loci further confirmed that both ligands are expressed in lamina neurons L3 and medulla neuron subtypes in likely overlapping patterns (Figures 3H and 3I).

The distribution of NetB protein was determined by Myc immunostaining in animals, in which *NetB* was replaced by C-terminal myc epitope-tagged *NetB* (*NetB^myc^*) cDNA using homologous recombination (Brankatschk and Dickson, 2006). In addition, protein localization was assessed in wild-type optic lobes labeled with NetB antisera (Albrecht et al., 2011) (Figures 3J–3M′ and S4). With both approaches, we detected NetB within the emerging M3 layer between R8 and R7 axons as early as 42 hr APF. NetB was highly concentrated within this layer at 55 hr APF. Expression decreased in adults. This spatial and temporal expression pattern within the M3 layer suggests that NetB could guide R8 axons to their recipient layer during the second targeting step.

### Netrin Is Required in the Target Area for Layer-Specific Targeting of R8 Axons

**Figure 4:**
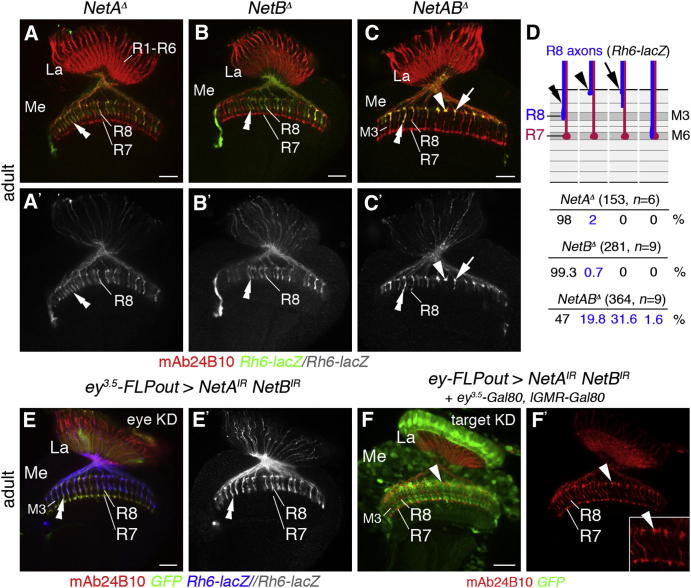
*NetB* Is Required in Target Neurons for R8 Axon Targeting to the M3 Layer (A–B′) In single mutants for *NetA^Δ^* and *NetB^Δ^*, *Rh6-lacZ*-positive R8 axons (green) target correctly to the M3 layer in the medulla (Me) neuropil (double arrowheads). (C and C′) In hemizygous *NetAB^Δ^* males, R8 axons stall at the medulla neuropil border (arrowheads) or terminate prematurely in layers M1/M2 (arrows) instead of M3 (double arrowheads). (D) Quantification of phenotypes (including the small percentage of axons mistargeting to the M6 layer) is presented. (E and E′) Knockdown (KD) of *NetA* and *NetB* in the eye using *UAS-RNAi* (*UAS-NetA^IR^* and *UAS-NetB^IR^*) and *ey^3.5^-FLP, act≫Gal4* FLPout transgenes: *Rh6-lacZ*-positive R8 axons (blue) correctly target to the M3 layer (double arrowheads). (F and F′) In flies, in which *NetA* and *NetB* have been knocked down in the target area using *ey-FLP, act≫Gal4* FLPout and *ey^3.5^-Gal80* and *lGMR-Gal80* transgenes, R8 axons stall at the medulla neuropil border (arrowheads) instead of terminating in M3 (double arrowheads). The inset shows R8 and R7 projections in the medulla at higher magnification. GFP (green) labels the cells in (E) and (F), in which *RNAi* transgenes are expressed. La, lamina; R cell axons are labeled with mAb24B10 (red). Scale bars, 20 μm.

If Netrins act as guidance cues that direct layer-specific targeting of Fra-expressing R8 axons, their loss in the target area should cause similar defects as that of *fra* in R cell axons. To test this prediction we examined *NetA^Δ^* and *NetB^Δ^* single as well as *NetAB^Δ^* double-mutant adult flies (Brankatschk and Dickson, 2006) (Figures 4A–4D). *Rh6-lacZ*-positive R8 axons targeted correctly to the M3 layer lacking either *NetA* (n = 6) or *NetB* (n = 9). However, in *NetAB^Δ^* double-mutant escapers, many R8 axons stalled at the medulla neuropil border (19.8%), terminated incorrectly in M1/M2 layers (31.6%), or proceeded to the deeper M6 layer (1.6%) (364 axons, n = 9). Thus, consistent with their expression pattern, *NetA* and *NetB* redundantly regulate layer-specific targeting of R8 axons. The penetrance of targeting defects observed in *NetAB^Δ^* homozygous mutants (53%) is lower than that detected in mosaic animals lacking *fra* function in the majority of R cells. This is in line with findings in the embryonic CNS by Yang et al. (2009) and may point toward a similar role of Fra in regulating the activity of additional guidance determinants. Knockdown of *NetA* and *NetB* in photoreceptor axons using RNAi transgenes did not cause any R8 axon-targeting errors (n = 10), while knockdown solely in the target resulted in similar defects as observed in *NetAB^Δ^* escapers (n = 12) (Figures 4E–4F′). Hence, Netrins are functionally required in neurons within the target area, but not in R cells.

### Ligand Capture and Local Release Control NetB Localization in Layer M3

**Figure 5:**
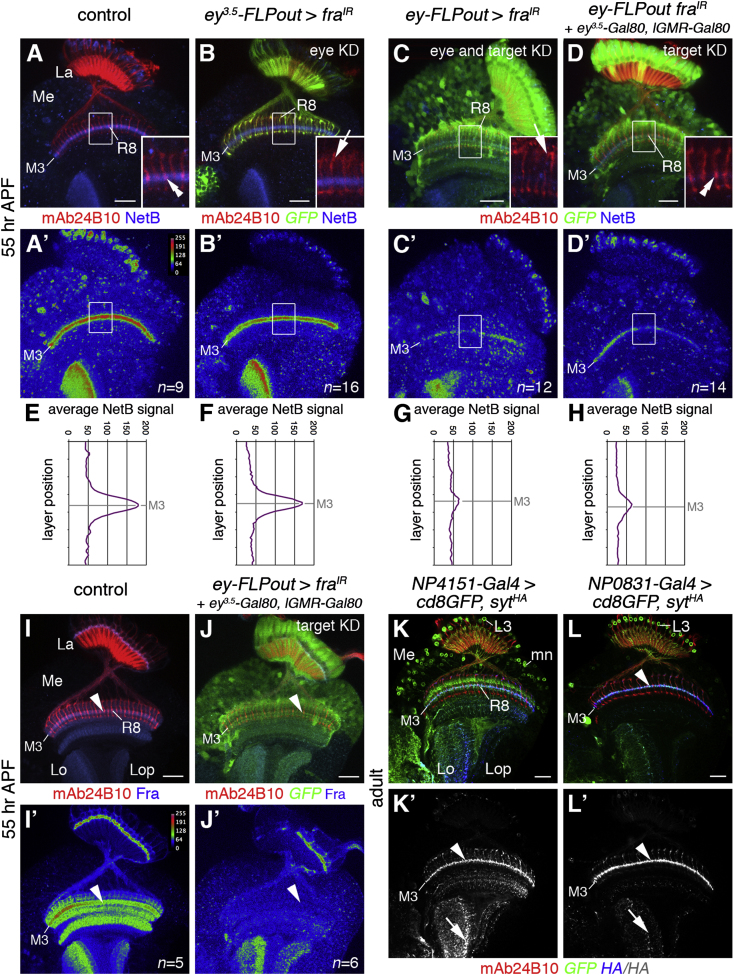
Ligand Capture and Local Release Contribute to Layer-Specific NetB Expression (A and A′) In controls at 55 hr APF, NetB (blue) is enriched in the M3 layer; R8 axons terminate in this layer (double arrowhead in inset in A). La, lamina; Me, medulla. (B and B′) *fra* knockdown (KD) in the eye using *ey^3.5^-FLP, act≫Gal4* FLPout transgenes: NetB localization in M3 is unaffected, despite the failure of R8 axons to extend into this layer (arrow in inset in B), suggesting that innervation by R8 axons is not required for maintenance of this layer. (C and C′) *fra* eye and target area knockdown using *ey-FLP, act≫Gal4* FLPout transgenes: NetB expression in M3 is strongly decreased, but not abolished; R8 axons show targeting defects (arrow in inset in C). (D and D′) *fra* target area knockdown using *ey-FLP, act≫Gal4* FLPout and *ey^3.5^-Gal80* and *lGMR-Gal80* transgenes: NetB expression in M3 is decreased; R8 axons target correctly (double arrowhead in inset in D). (E–H) Graphs present average NetB fluorescence intensities within white boxes (A–D′). (I and I′) In controls, Fra (blue) is widely expressed in the medulla including the M3 layer (arrowheads). (J and J′) *fra* target area knockdown using *ey-FLP, act≫Gal4* FLPout and *ey^3.5^-Gal80* and *lGMR-Gal80* transgenes: Fra expression is strongly decreased in the medulla neuropil including the M3 layer (arrowheads). (K–L′) *NP4151*- and *NP0831-Gal4* drive expression of the presynaptic marker *UAS-syt^HA^* (blue) and GFP (green). Primary release sites of NetB-expressing neurons include the axon terminals of lamina neurons L3 in layer M3 (arrowheads) and of medulla neurons in the lobula (arrow, Lo). Lop, lobula plate. R cell axons are labeled with mAb24B10 (red). Fra or NetB protein expression is shown in pseudocolor in (A′), (B′), (C′), (D′), (I′), and (J′) (see scale for values). GFP expression (green) marks the cells in (B), (C), (D), and (J), in which *UAS-fra^IR^* is expressed. Scale bars, 20 μm. See also Figure S5.

Despite being a diffusible ligand, NetB is highly enriched in a narrow layer. How is such localized distribution achieved? Fra has been shown to capture and relocalize midline-derived Netrins along dorso-lateral regions within the embryonic CNS (Hiramoto et al., 2000). Therefore, we tested the ability of target-derived Fra to influence NetB or NetB^myc^ distribution (Figures 5A–5H and S5A–S5C″). Knockdown of *fra* in the eye by combining the FLPout approach and a *ey^3.5^-FLP* transgene did not have any effect (n = 16). However, knockdown in the eye and target area using the FLPout approach in conjunction with *ey-FLP* (n = 12) or solely the target area using additional *ey^3.5^-Gal80* and *lGMR-Gal80* transgenes (n = 14) resulted in a considerable reduction of NetB in the M3 layer. Furthermore, optic lobes of flies, in which *fra* has been knocked down in the eye and in the target area, were labeled with Fmi as an independent M3 layer marker. At 55 hr APF, Fmi expression in this layer was unaffected, while the NetB signal was reduced compared to controls (Figures S5D–S5E″), excluding the possibility that expression is decreased because the M3 layer failed to form.

Although *fra* was significantly reduced (Figures 5I–5J′), layer-specific distribution of NetB was not completely abolished (Figures 5C′ and 5D′), suggesting that this expression could be attributed to local ligand release. To determine the main output areas, we expressed HA-tagged Synaptotagmin (Chou et al., 2010) within the NetB-positive neuron population using *NP4151-Gal4* and *NP0831-Gal4* (Figures 5K–5L′). We observed strong expression in the M3 layer, which overlapped with the axon terminals of lamina neurons L3 and resembled the distribution of NetB protein during midpupal development. Furthermore, we detected increased Synaptotagmin expression in the lobula, likely originating from Tm neurons, consistent with our observation that NetB is also strongly expressed in the lobula neuropil (Figures 3J–3L′). These findings suggest that primarily axon terminals rather than dendrites release Netrins. Hence, two mechanisms contribute to the localization of Netrins in the M3 layer: (i) local release by lamina neuron L3 axon terminals, and (ii) capture by target neuron-associated Fra to reduce diffusion.

### NetB Functions as Short-Range Guidance Cue

**Figure 6:**
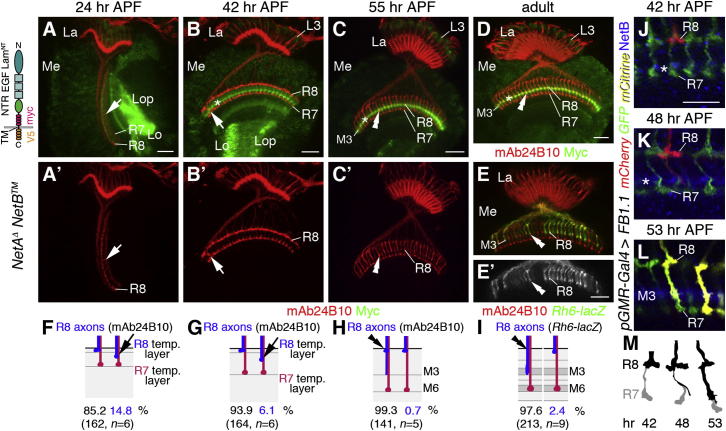
*NetB* Acts at Short Range (A–E′) Membrane-tethered myc-tagged *NetB* (green indicates *NetB^TM^* expression in A, B, C, and D) replaces endogenous *NetB* in a *NetA^Δ^* mutant background. Schematic of NetB^TM^ shows the laminin N-terminal domain (Lam^NT^), EGF repeats, Netrin-like domain (NTR), transmembrane domain (TM), and myc and V5 tags. *NetB^TM^* is expressed by lamina neurons L3 and medulla neurons (mn) and enriched in the emerging and final M3 layer (asterisks in B, C, and D). At 24 and 42 hr APF, few R8 axons extend incorrectly past their temporary layer (arrows in A–B′). At 55 hr (C and C′) and in adults (D–E′), R8 axons labeled with mAb2B10 (red) and *Rh6-lacZ* (green in E and E′) correctly terminate in M3 (double arrowheads in C–E′). (F–I) Quantification of phenotypes is shown. (J–L) R8 axons were labeled with *pGMR-Gal4* and *FB1.1*. At 42 hr, R8 growth cones spread within the temporary layer (J), at 48 hr, they extend a thin filopodium (arrowhead) along R7 axons toward the emerging M3 layer (asterisk in K), and at 53 hr, this process develops into a mature terminal (L). (M) Drawing shows changing R8 growth cone morphology during pupal development. La, lamina; Me, medulla; Lo, lobula; Lop, lobula plate. R cells are labeled with mAb24B10 (red in A–E). Scale bars, 20 μm (A–E′) and 10 μm (J–L).

Netrins act as short-range attractants at the embryonic midline of *Drosophila* (Brankatschk and Dickson, 2006). We therefore tested whether Netrins could act in a similar manner within the visual system by examining R cell projections of flies, which have been modified by homologous recombination to solely express membrane-tethered NetB (*NetB^TM^*) at near-endogenous levels (Brankatschk and Dickson, 2006). We observed that membrane-bound NetB was strongly enriched in the emerging M3 layer. While a small percentage of R8 axons abnormally projected past the distal medulla neuropil border at 24 and 42 hr APF (n = 6 for each stage), projections were unaffected at 55 hr (n = 5) and in adults (n = 9) (Figures 6A–6I). In flies in which *fra* has been knocked down in the target area, NetB^TM^ levels remained high, further supporting the notion that target-associated Fra prevents soluble NetB from diffusion (Figures S5F and S5F′). Together, these findings suggest that target layer recognition of R8 axons depends on locally acting Netrins in layer M3.

Furthermore, we examined the morphology of single R8 growth cones using the *Flybow FB1.1* approach (Hadjieconomou et al., 2011a) in conjunction with the R cell-specific *pGMR-Gal4* driver during pupal development (Figures 6J–6M). At 42–44 hr APF (n = 11), R8 growth cones spread along the distal medulla neuropil border as they pause in their temporary layer. At 48–50 hr (n = 19), they extended a single thin filopodium along the R7 axon shaft toward the NetB-positive emerging M3 layer. At 52–55 hr (n = 7), the growth cone core at the medulla neuropil border was decreased in size, while the filopodium increased in thickness to eventually develop into a mature terminal. Thus, filopodial extensions of R8 axons could bridge the distance to the NetB-positive layer M3 to mediate short-range interactions.

### NetB Expression in Lamina Neurons L3 Rescues R8 Axon-Targeting Defects

**Figure 7:**
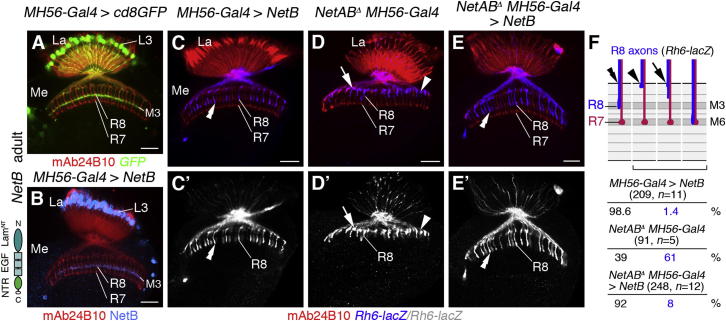
*NetB* Expression in Lamina Neurons L3 Rescues R8 Axon-Targeting Defects (A) *MH56-Gal4* drives GFP expression (green) in adult lamina (La) neurons L3. (B) NetB (blue) is detected in cell bodies of lamina neurons L3 and in their axon terminals in layer M3 of adults upon expression with *MH56-Gal4*. Schematic of NetB protein shows the laminin N-terminal domain (Lam^NT^), EGF repeats, and Netrin-like domain (NTR). (C and C′) Overexpression of *NetB* with *MH56-Gal4* does not interfere with targeting of *Rh6-lacZ*-positive R8 axons (blue, double arrowheads). (D and D′) In hemizygous *NetAB^Δ^* males, many *Rh6-lacZ*-positive R8 axons stall at the medulla (Me) neuropil border (arrowheads) or prematurely terminate in layers M1/M2 (arrows). (E and E′) Expression of *UAS-NetB* with *MH56-Gal4* rescues R8 axon-targeting defects of *NetAB^Δ^* mutants. R8 axons target correctly to layer M3 layer (double arrowheads). (F) Quantification of phenotypes is shown. Lo, lobula; Lop, lobula plate. R cells are labeled with mAb24B10 (red in A–C, D, E, and F; white in C′, D′, and E′). Scale bars, 20 μm. See also Figure S6.

To test whether lamina neurons L3 indeed provide the local Netrin signal, we next conducted rescue experiments using *MH56-Gal4*, a driver with strong activity in lamina neurons L3 throughout pupal development (Figures 7A and S6). Overexpression of NetB with *MH56-Gal4* did not interfere with R8 axon targeting (Figures 7B–7C′). Strikingly, expression of NetB in a *NetAB^Δ^* background significantly rescued R8 axon-targeting defects (Figures 7D–7F). Only 8% of *Rh6-lacZ*-expressing neurons stalled at the medulla neuropil border or terminated in the distal M1/M2 layers (248 axons, n = 12) compared to 61% in mutant siblings lacking *UAS-NetB* (91 axons, n = 5). While we cannot exclude a contribution of other neuron subtypes, these findings indicate that NetB in lamina neurons L3 is sufficient to control layer-specific targeting of R8 growth cones.

### NetB Is Instructive for R8 Axon Targeting

**Figure 8:**
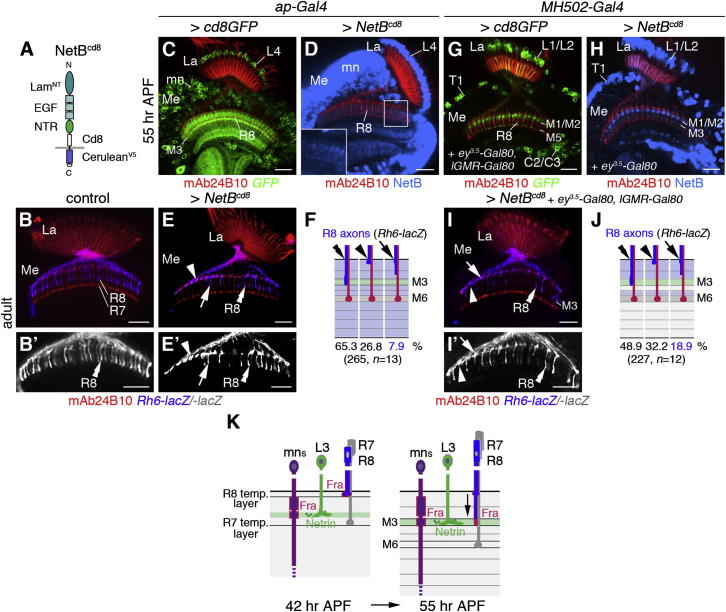
Localized NetB Is Sufficient to Target R8 Axons to Ectopic Layers (A) NetB^cd8^ schematic showing laminin N-terminal (Lam^NT^), EGF, Netrin-like (NTR), Cd8, and V5-epitope-tagged Cerulean domains. (B and B′) In controls, *Rh6-lacZ*-positive R8 axons (blue) terminate in layer M3 (double arrowheads). (C–F) Expression is under the control of *ap-Gal4*. At 55 hr APF, GFP (green in C) and NetB^cd8^ (blue in D) are detected in cell bodies and processes of lamina (La) neurons L4 and medulla (Me) neurons (mn). In adults, many R8 axons stop at the medulla neuropil border (26.8%, arrowheads) and in distal layers (7.9%, arrows) (E and E′). Quantification of phenotypes is shown in (F). (G–J) Expression is under the control of *MH502-Gal4*. GFP labels lamina neurons L1/L2, T1 medulla, and C2/C3 neurons (G). NetB^cd8^ localizes to layers M1/M2 (H). Of R8 axons, 18.9% terminated in M1/M2 (arrows); 32.2% stalled at the border (arrowheads) (I and I′). Quantification of phenotypes is shown in (J). In (F) and (J), green symbolizes endogenous and blue ectopic NetB expression. (K) This model illustrates that during early development, axon terminals of lamina neurons L3 locally release NetB in the emerging M3 layer. Fra expressed by unidentified medulla neurons captures NetB. Fra-positive R8 axons pause in their temporary layer. During midpupal development, R8 axons expressing Fra are guided to the NetB-positive M3 layer. Scale bars, 20 μm. See also Figure S7.

Finally, we tested whether layer-specific localized Netrins could play an instructive role in controlling R8 axon targeting. For this purpose we assessed the effects of ectopically expressing membrane-tethered NetB using a *UAS-NetB^cd8^* transgene (Figure 8A) under two conditions. First, NetB^cd8^ was widely expressed in medulla neuropil layers using *ap-Gal4*, a driver active in lamina neurons L4 and in 40% of medulla neurons (Morante and Desplan, 2008). Unlike in controls, 27% of *Rh6-lacZ*-positive R8 axons stalled at the medulla neuropil border, while 8% terminated incorrectly in the M1/M2 layers (265 axons, n = 13) (Figures 8B–8F). Second, NetB^cd8^ was directed to a subset of ectopic layers using *MH502-Gal4*, a driver active in lamina neurons L1/L2, ascending T1 medulla neurons and C2/C3 neurons throughout development. Transient expression in R cells during larval and pupal development was suppressed using *ey^3.5^-Gal80* and *lGMR-Gal80* transgenes (Figures 8G and S7). Expression analysis at 55 hr confirmed that high levels of NetB are present in layers M1/M2 (Figure 8H). Despite the presence of endogenous Netrins in the M3 layer, 32% of *Rh6-lacZ*-positive R8 axons stalled at the medulla neuropil border, while 19% stopped in the M1/M2 layers (227 axons, n = 12) (Figures 8I–8J). Using *NP1086-Gal4* (Rister et al., 2007), we also expressed NetB^cd8^ in T1 neurons, which extend dendrites into layer M2 and axons into the lamina. Membrane-tethered ligand was not detected in the medulla, but in the lamina, and consistently, R8 axon targeting to layer M3 was unaffected (Figures S7L–S7M′). This confirms that axons are the primary site of NetB release, and ectopic ligand expression using *MH502-Gal4* can be mainly attributed to lamina neurons L1 and L2. The increased percentage of redirected axons to defined NetB-expressing layers with *MH502-Gal4* compared to the effects of wide ectopic expression using *ap-Gal4* supports the model that layer-specific localization of Netrins is sufficient for R8 axon targeting.

## Discussion

### A Role for Netrins and Fra in Layer-Specific Axon Targeting

Recent studies identified at least four molecular mechanisms that control layer-specific targeting in the nervous system by cell-cell interactions independently of neural activity. First, combinatorial expression of homophilic cell surface molecules promotes the recognition and stabilization of contacts between matching branches of pre- and postsynaptic neuron subsets. For instance, four members of the immunoglobulin superfamily of cell adhesion molecules, Sidekick 1 and 2 and Dscam and DscamL, are expressed and required in subsets of bipolar, amacrine, and retinal ganglion cells for targeting to different inner plexiform sublayers (IPLs) in the chick retina (Yamagata and Sanes, 2008). In *Drosophila*, the leucine-rich repeat protein Caps may play an analogous role, as it is specifically expressed in R8 cells and target layers M1–M4 and, thus, could promote homophilic interactions to stabilize connections within correct columns and layers (Shinza-Kameda et al., 2006). Second, concise temporal transcriptional control is used to regulate the levels of ubiquitous cell surface molecules and, thus, adhesiveness of afferent and target neurons to balance branch growth and targeting. This mechanism is supported by findings in the fly visual system where the transcription factor Sequoia controls R8 and R7 axon targeting by the temporal regulation of N-Cadherin (CadN) expression levels (Petrovic and Hummel, 2008). Third, repellent guidance cues are utilized to exclude projections from some layers, as has been shown for membrane-bound Semaphorin family members and Plexin receptors in the IPL of the mouse retina (Matsuoka et al., 2011a, 2011b). Fourth, recent studies also implicated the graded expression of extracellular matrix-bound guidance cues such as Slit in the organization of layered connections in the zebrafish tectum (Xiao et al., 2011). Our findings for the essential role of Netrins and Fra in visual circuit assembly provide evidence for a different strategy: a localized chemoattractant guidance cue is used to single out one layer, thus providing precise positional information required for layer-specific axon targeting of cell types expressing the receptor. Unlike in the ventral nerve cord, where the Netrin/Fra guidance system controls growth across the midline (Brankatschk and Dickson, 2006; Dickson and Zou, 2010), in the visual system, it mediates target recognition by promoting axon growth into but not past the Netrin-positive layer.

### A Role for Lamina Neurons L3 as Intermediate Targets

Our rescue experiments support the model that Netrins are primarily provided by the axon terminals of lamina neurons L3 in the M3 layer. During early pupal stages, Fra-positive R8 axons pause in their temporary layer at the distal medulla neuropil border. From midpupal development onward, upon release from this block, Fra-positive R8 axons are guided to the Netrin-expressing M3 layer (Figure 8K).

Axons can use intermediate target cells either along their trajectory to guide them toward their target areas or within the target area to bring putative synaptic partners into close vicinity (Sanes and Yamagata, 2009). Although R8 axons and lamina neurons L3 terminate closely adjacent to each other in the same layer, they have been described to not form synaptic connections with each other but to share common postsynaptic partners such as the transmedullary neuron Tm9 (Gao et al., 2008; Takemura et al., 2008). Thus, our results suggest that layer-specific targeting of R8 axons relies on the organizing role of lamina neurons L3 as intermediate targets in the M3 layer rather than direct interactions with postsynaptic partners. Consistent with this notion, axons of lamina neurons L3 timely extend between the temporary layers of R8 and R7 axons from early pupal stages onward, and targeting of their axons is independently controlled by other cell surface molecules such as CadN (Nern et al., 2008). Further studies will need to identify potential Fra-positive synaptic partners in the medulla and test whether this guidance receptor equally controls targeting of their dendritic branches, thus bringing pre- and postsynaptic neurites into the same layer. Additional mechanisms likely mediate cell-cell recognition and synaptic specificity, as electron microscopic analysis showed that presynaptic sites in R8 axons are not restricted to the M3 layer but distributed along the axon (Takemura et al., 2008).

### Local Release and Receptor-Mediated Capture of Netrins

Netrins are diffusible guidance cues acting both at long range in a gradient and at short range when immobilized (Lai Wing Sun et al., 2011). Consistent with studies in the *Drosophila* embryo (Brankatschk and Dickson, 2006), we observed that NetB in the visual system acts at short range, as R8 axon targeting is normal when solely membrane-tethered NetB is available at near-endogenous levels. Secreted Netrins are converted into a short-range signal because they are locally released by lamina neurons L3 and prevented to diffuse away through a Fra-mediated capturing mechanism. Filopodial extensions could enable R8 growth cones to bridge the distance to NetB-expressing lamina neuron L3 axon terminals.

Although in principle Netrins could be secreted by both dendritic and axonal arbors of complex neurons, our results support the notion that axon terminals are the primary release sites to achieve layer-specific expression. This may be mediated by a cargo transport machinery along polarized microtubules similar to that used by synaptic proteins or neurotransmitters (Rolls, 2011). Consistently, recent findings in *C. elegans* identified proteins involved in motor cargo assembly and axonal transport as essential for Netrin localization and secretion (Asakura et al., 2010). Intermediate target neurons may thus constitute an important strategy to draw afferent axons into a layer, if guidance cues are preferentially released by axon terminals and not by dendritic branches of synaptic partner neurons. Netrin-releasing lamina neurons L3 form dendritic spines in the lamina and axon terminals in the medulla. Similarly, Netrin-positive transmedullary neuron subtypes such as Tm3 and Tm20 form dendritic branches in the medulla and extend axons into the lobula. Thus, a mechanism, whereby neurons in one brain area organize the connectivity in the next, may be used at least twice in the visual system.

Knockdown of *fra* in the target area strongly reduced NetB in the M3 layer, supporting the notion that a receptor-mediated capturing mechanism controls layer-specific Netrin accumulation. Despite the use of multiple genetic approaches, we did not observe R8 axon-targeting errors when manipulating Fra levels exclusively in target neurons (Figure 5). This could be attributed to the technical limitation that knockdown is incomplete owing to the activity of the *ey* enhancer in around 50% of medulla neurons (Morante and Desplan, 2008). However, as lamina neurons L3 continue to locally release Netrins, remaining ligands may likely be sufficient to guide fully responsive R8 axons to their target layer.

Unlike in the fly embryonic CNS, where Netrins are captured by Fra and presented to growth cones expressing a Netrin receptor other than Fra (Hiramoto et al., 2000), or in *C. elegans*, where Unc-6 is captured at the dendrite tips of nociceptive neurons by Unc-40 to interact with Unc-5 (Smith et al., 2012), our genetic analyses indicate that *fra* is required in R8 axons. Hence, Netrins captured by Fra-positive target neurons may either be presented to Fra-expressing R8 axons in a dynamic fashion, or R cell- and target neuron-derived Fra interact with Netrins in a ternary complex in *trans*. This is conceivable since (1) the vertebrate counterpart Netrin-1 shows a high binding affinity for DCC (K_d_ = 10^−8^ M) (Keino-Masu et al., 1996); (2) DCC can bind Netrins with multiple domains (DCC, fourth and fifth fibronectin type III domains; Netrins, Laminin N-terminal (Lam^NT^) and three Laminin-type epidermal growth factor [EGF]-like domains) (Geisbrecht et al., 2003; Kruger et al., 2004); and (3) at least in *cis*, Netrins can bind and aggregate multiple DCC ectodomain molecules (Stein et al., 2001). Ligand capture and presentation by receptors have also been reported for F-spondin and lipoprotein receptor-related protein (LRP) at the vertebrate floor plate (Zisman et al., 2007). Netrins have previously been shown to promote exocytosis and recruitment of their receptor to distinct subcellular locations on cell surfaces (Adler et al., 2006; Matsumoto and Nagashima, 2010). Moreover, in the visual system, Netrins may increasingly draw neurites of Fra-positive target neurons into layer M3, which in turn could promote further ligand accumulation. Thus, additional feedback loops may contribute to the specific enrichment of both Netrins and Fra in the M3 layer.

### An Instructive Role for Netrins in Directing R8 Axons to Their Recipient Layer

R8 axon targeting involves multiple successive steps (Hadjieconomou et al., 2011b): (1) the selection of the retinotopically correct column; (2) pausing in the temporary layer; (3) timely release from the temporary layer and extension of a filopodium; (4) bypassing of incorrect neuropil layers; (5) correct identification and targeting to the M3 layer; (6) stabilization of connections in the correct layer and column and transformation of growth cones into mature terminals; and (7) formation of the correct repertoire of synaptic contacts. Strong early defects would likely impact on subsequent steps.

Within this sequence of events, interactions of Gogo and Fmi in *cis* within R8 axons and in *trans* with Fmi-positive neuronal processes in the emerging M1, M2, and lower M3 layers have been shown to contribute to the timely release of R8 growth cones from their temporary layer and, consequently, enable correct targeting to the M3 layer (Hakeda-Suzuki et al., 2011; Mann et al., 2012; Tomasi et al., 2008) (steps 3 and 6). Caps may specifically promote cell-cell recognition and stabilize interactions between R8 axons and target neuron branches within their correct column and target layer (Shinza-Kameda et al., 2006) (step 6). However, an alteration of adhesiveness may not be sufficient to promote the extension of filopodia toward the correct layer, and additional attractive guidance forces are required. The Netrin/Fra guidance system is well suited to play such a role by providing the necessary positive forces directing filopodia toward deeper layers and by promoting recognition of a single layer at a given position (steps 4 and 5). This notion is supported by our observations that loss of Fra or Netrins causes many R8 axons to stall at the distal medulla neuropil border and to terminate at interim positions in layers M1/M2. Furthermore, ectopic expression of membrane-tethered NetB is sufficient to retarget a significant proportion of R8 axons. Unlike Caps and Gogo/Fmi, whose ectopic expression can promote targeting of some R7 axons to the M3 layer (Hakeda-Suzuki et al., 2011; Shinza-Kameda et al., 2006), Fra was not sufficient to redirect R7 axons from the M6 to the M3 layer. A likely explanation is that the effects of R7-specific guidance determinants cannot be overwritten, or essential cooperating receptors or downstream components of Fra present in R8 are missing in R7 cells. Furthermore, overexpression of Fra causes many R8 axons to stall at the medulla neuropil border, suggesting that tight temporal regulation of receptor levels in R8 axons is essential for the integration of an additional potential repellent input.

Together, these findings in the *Drosophila* visual system suggest that the dynamic coordinated actions of chemotropic guidance cues and cell adhesion molecules contribute to layer-specific targeting of specific cell types. A similar molecular mechanism relying on Netrins or other localized attractive guidance cues and their receptors may be more widely used for the assembly of laminated circuits.

## Experimental Procedures

### Molecular Cloning

*pUAS-fra^IR^*, *pUAS-NetB^IR^*, and *UAS-NetB^cd8^* constructs were generated using standard cloning techniques. For details see Supplemental Experimental Procedures.

### Genetics

Functional analyses were conducted using combinations of the *Gal4/UAS* system (Brand and Perrimon, 1993), the *FLP/FRT* system-based *ey-FLP* (Newsome et al., 2000), *ey^3.5^-FLP* (Bazigou et al., 2007), MARCM (Lee and Luo, 1999), Flybow (Hadjieconomou et al., 2011a), and FLPout (Ito et al., 1997) techniques, as well as *UAS-RNAi*-based approaches (Dietzl et al., 2007). Gal4 activity was specifically suppressed in R cells using the transgenes *ey^3.5^-Gal80* (Chotard et al., 2005) and *lGMR-Gal80* (kindly provided by C. Desplan). A comprehensive description of parental stocks and crosses, experimental conditions, as well as full genotypes of samples shown in main and supplemental figure panels is provided in Supplemental Experimental Procedures and Tables S1 and S2.

### Histology and Imaging

Brains were dissected in PBS, fixed for 1 hr in 2% paraformaldehyde (PFA) in 0.1 M L-lysine containing 0.05 M phosphate buffer, and washed in PBS containing 0.5% Triton X-100 (Sigma-Aldrich). For immunolabeling of brains, the following primary antibodies were used: mouse mAb24B10 (1:75; Developmental Studies Hybridoma Bank [DSHB]); rabbit anti-β-galactosidase (1:12,000; Cappel); rabbit anti-Fra (1:200; Kolodziej et al., 1996); rat anti-HA (1:500; Roche); rabbit anti-Myc (1:250; Santa Cruz Biotechnology); rabbit anti-NetB (1:500; Albrecht et al., 2011); and mouse anti-V5 (Invitrogen, 1:500). For immunofluorescence analyses, the following secondary antibodies were used: goat anti-mouse, rabbit, and rat F(ab′)_2_ fragments coupled to FITC (1:200), Cy3/DyLight549 (1:400) or Cy5/DyLight649 (1:200) (Jackson ImmunoResearch Laboratories), as well as goat anti-mouse Alexa Fluor 568 (1:400; Invitrogen). As the V5 epitope was detectable using anti-V5 antibody in western blots but not in cells or tissues, NetB was visualized using anti-NetB antibody. Images were collected using Zeiss/Bio-Rad Radiance2100, Leica TCS SP5II, and Zeiss LSM710 laser-scanning confocal microscopes. Immunofluorescence levels were determined using ImageJ; neurons were traced using Fiji Simple Neurite Tracer. For stainings shown in supplemental figures, see Supplemental Experimental Procedures. Detailed staining protocols are available upon request.

## Supplemental Information

Document S1. Figures S1–S7, Tables S1 and S2, and Supplemental Experimental Procedures
