# Supplementary material for: Localized Netrins Act as Positional Cues to Control Layer-Specific Targeting of Photoreceptor Axons in Drosophila
Source: Neuron. 2012 Jul 12;75(1):80–93. doi: 10.1016/j.neuron.2012.04.037 (PMC3398394; doi:10.1016/j.neuron.2012.04.037)
Supplement: Document S1. Figures S1–S7, Tables S1 and S2, and Supplemental Experimental Procedures [file mmc1.pdf]

Neuron, Volume *75*

Supplemental Information

Localized Netrins Act as Positional Cues to Control Layer-Specific Targeting of Photoreceptor Axons in *Drosophila*

Katarina Timofeev, Willy Joly, Dafni Hadjieconomou, and Iris Salecker

SUPPLEMENTAL FIGURES AND LEGENDS

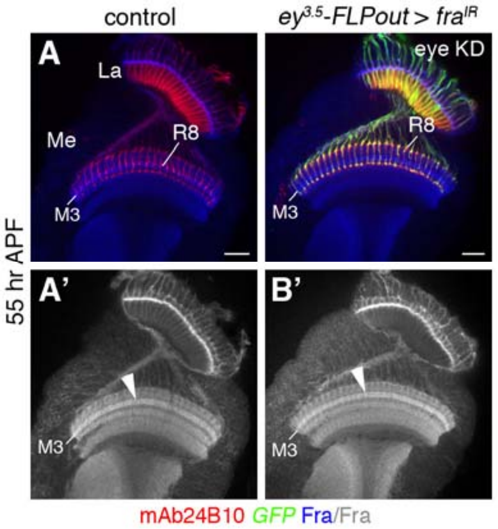

Figure S1, related to Figure 1. Fra Expression in Target Neurons
(A-B') Comparison of flies, in which *fra* has been knocked-down in R-cells using *ey*$^{3.5}$-*FLP*, *act>>Gal4* FLPout transgenes (B, B', *n*=14), with control siblings (A, A', *n*=8) confirms that the Fra receptor (blue) is widely expressed in target neurons and enriched in the M3 layer (arrowhead) at 55 hr after puparium formation (APF). R8 axons fail to innervate the M3 layer and stall at the medulla neuropil border (see also Figure 2). R-cells are labeled with mAb24B10 (red). GFP (green) labels the cell types, in which the *UAS-fra*^{IR} transgene is expressed. La, lamina; Me, medulla.
Scale bar, 20 μm.

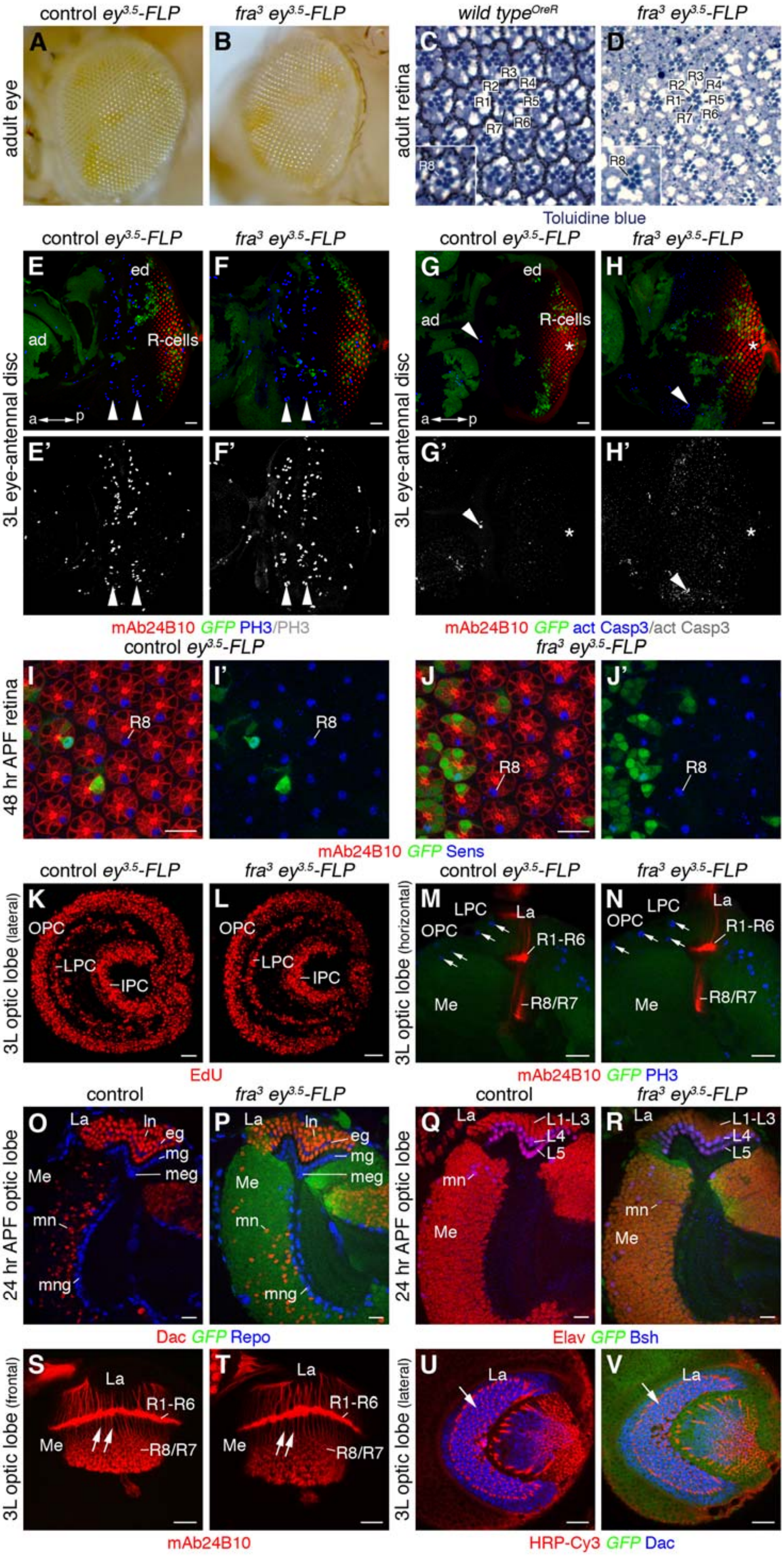

control ey3.5-FLP
fra3 ey3.5-FLP
wild typeOreR
fra3 ey3.5-FLP
adult eye
adult retina
A
B
C
D
R1 R2 R3 R4 R5 R6 R7 R8
Toluidine blue
E F G H
E' F' G' H'
ed
ad
R-cells
a p
3L eye-antennal disc
mAb24B10 GFP PH3/PH3
mAb24B10 GFP act Casp3/act Casp3
48 hr APF retina
I I' J J'
mAb24B10 GFP Sens
K L M N
OPC LPC IPC La Me
R1-R6
R8/R7
3L optic lobe (lateral)
3L optic lobe (horizontal)
EdU
mAb24B10 GFP PH3
control
24 hr APF optic lobe
O P Q R
ln eg mg meg mn mng
L1-L3 L4 L5
Dac GFP Repo
Elav GFP Bsh
S T U V
3L optic lobe (frontal)
mAb24B10
HRP-Cy3 GFP Dac

Figure S2, related to Figure 2. R-cells, Target Neurons and Glia Develop Normally in *fra*^{3} *ey*$^{3.5}$-*FLP* Mosaics

(A and B) As in controls (A), eyes of *fra*^{3} *ey*$^{3.5}$-*FLP* mosaic adult flies (B) show a smooth array of ommatidia within large clones of homozygous mutant cells. Clones were identified based on reduced *mini-white* transgene expression and pigmentation. This reduction was more pronounced in *fra*^{3} mosaic eyes.

(C and D) Analysis of toluidine blue-stained semithin sections of adult eyes shows that ommatidia of *fra*^{3} *ey*$^{3.5}$-*FLP* mosaics (D) are indistinguishable from wild type (C) and display a normal complement and trapezoid arrangement of R-cell bodies with healthy rhabdomeres, indicating that adult R-cell specification and survival is normal (wild type: 563/563 ommatidia, *n*=5; *fra*^{3} *ey*$^{3.5}$-*FLP*: 419/426 ommatidia, 98.4%, *n*=5). Insets show sections taken at the R8 level.

(E-F') Confocal images of 3rd instar larval (3L) eye-antennal discs labeled with an antibody against phospho Histone 3 (PH3, blue) show that proliferation of R-cells within two mitotic waves (arrowheads), as well as their differentiation within clusters and rows occurs normally in *fra*^{3} *ey*$^{3.5}$-*FLP* mosaics (F, F', *n*=23) compared to controls (E, E', *n*=21).

(G-H') *fra*^{3} mutant eye discs (H, H', *n*=3) do not show increased levels of activated Caspase3 staining (blue, arrowheads) within the eye field compared to controls (G, G', *n*=3), suggesting that cell survival is not significantly affected using this allele. ad, antennal disc; ed, eye disc.

(I-J') Labeling of the pupal retina at 48 hr after puparium formation (APF) with an antibody against Senseless (Sens, blue) confirms that compared to controls (I, I', *n*=4) R8 cell specification in *fra*^{3} *ey*$^{3.5}$-*FLP* mosaics (J, J', *n*=6) is unaffected.

R-cells are labeled with mAb24B10 (red, E, F, G, H, I, J), Areas lacking GFP expression (green) correspond to control or *fra*^{3} mutant cells.

(K and L) 3rd instar larval optic lobes incubated with EdU (red) to visualize proliferation zones show indistinguishable staining within the outer proliferation center (OPC), lamina precursor cell (LPC) area and inner proliferation center (IPC) in control (K, *n*=17) and *fra*^{3} *ey*$^{3.5}$-*FLP* mosaics (L, *n*=13).

(M and N) 3rd instar larval optic lobes labeled with an antibody against phospho Histone H3 (PH3, blue) to visualize cells in mitosis within the OPC and the LPC area show similar cell division patterns (arrows) in control (M, *n*=25) and *fra*^{3} *ey*$^{3.5}$-*FLP* mosaics (N, *n*=14).

(O-R) Labeling with differentiation markers shows that general neuronal and glial development is not affected at this level of resolution by loss of *fra*^{3} in R-cells at 24 hr APF. As in control siblings (O, *n*=6), lamina neurons (ln) in *fra*^{3} *ey*$^{3.5}$-*FLP* mosaics (P, *n*=5) are incorporated into columns and express the early differentiation marker Dachshund (Dac, red); epithelial (eg), marginal (mg) and medulla (meg), as well as medulla neuropil (mng) glial cells express the differentiation marker Reversed polarity (Repo, blue) and have migrated to their characteristic positions above and below the lamina plexus, and adjacent to the medulla neuropil, respectively. Lamina neurons (L1-L5) and medulla neurons (mn) express the late differentiation markers Elav (Embryonic lethal abnormal vision, red), and lamina neurons L4 and L5, and some medulla neurons (mn) express the cell-fate specific marker Brain-specific homeobox protein (Bsh, blue) in controls (Q, *n*=8) and *fra*^{3} *ey*$^{3.5}$-*FLP* mosaics (R, *n*=5).

(S and T) Frontal view of 3rd instar larval optic lobe: the R-cell projection pattern visualized with mAb24B10 (red) is normal in *fra*^{3} *ey*$^{3.5}$-*FLP* mosaics (T, *n*=4) compared to controls (S, *n*=3). R1-R6 growth cones correctly terminate within the lamina plexus, and R8/R7 axon bundles form a regular array of evenly spaced bundles (arrows) and growth cones within the medulla neuropil.

(U and V) Lateral view of 3rd instar larval optic lobe: the spacing of R-cell bundles labeled with horseradish peroxidase antibody (HRP, red) within the lamina is normal in *fra*^{3} *ey*$^{3.5}$-*FLP* mosaics (V, *n*=6) compared to controls (U, *n*=6). Lamina neurons were labeled with Dac (blue). La, lamina; Me, medulla. Mosaic animals express *Ubi-GFP* in the target area, control siblings lack this expression.

Scale bars, 20 μm.

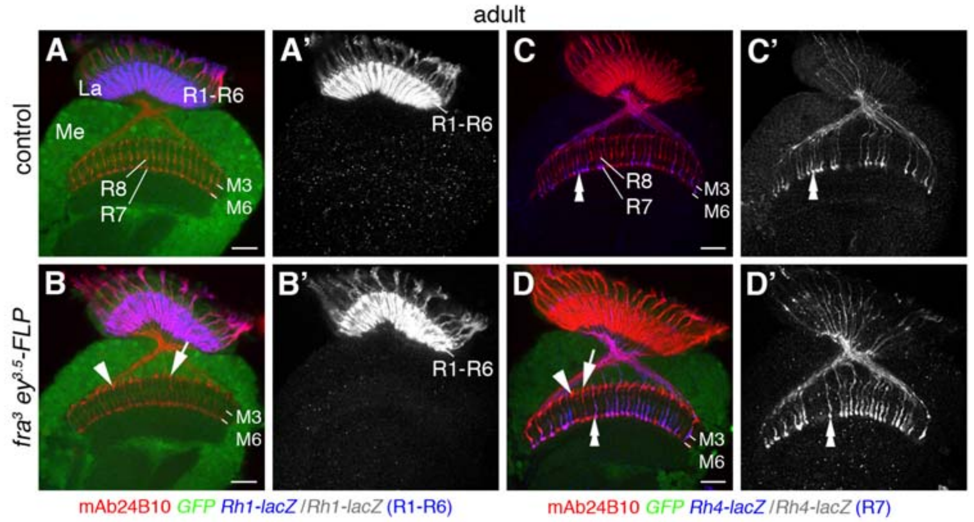

Figure S3, related to Figure 2. Ganglion-specific Targeting of R1-R6 axons and Layer-specific Targeting of R7 axons is not Affected by Loss of *fra*

(A-B') As in controls (A, A', *n*=21), *Rh1-lacZ* positive R1-R6 axons (blue) correctly terminate within the lamina (La) in *ey*$^{3.5}$*-FLP* mosaic animals lacking *fra* in R-cells (B, B', *n*=5), indicating that ganglion-specific targeting is unaffected.

(C-D') As in controls (C, C'), *Rh4-lacZ* positive R7 axons (double-arrowheads, blue) correctly terminate in layer M6 within the medulla (Me) in *fra*^{3} *ey*$^{3.5}$*-FLP* mosaic animals (D, D', 100%, 282/282 *Rh4-lacZ* positive R7 axons, *n*=9). *fra* mutant R8 axons stall at the medulla neuropil border (arrowheads) or prematurely terminate in layers M1/M2 (arrows). R-cells are labeled with mAb24B10 (red, A, B, C, D).

Scale bars, 20 µm.

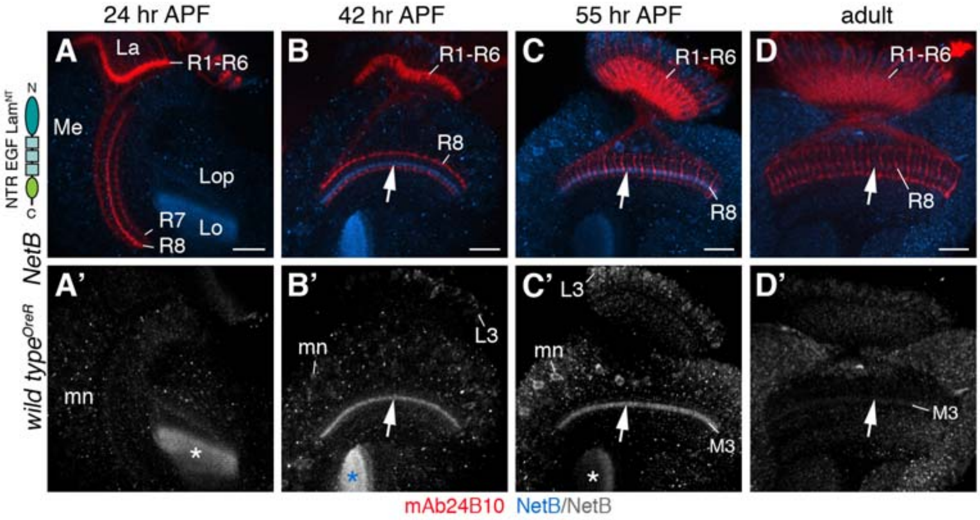

Figure S4, related to Figure 3. NetB Protein is Localized in the M3 Layer

(A-D') Immunolabeling of wild type optic lobes during pupal development and in adults with a NetB antibody (blue) reveals a similar expression pattern as Myc labeling of *NetA*$^{\Delta}$ *NetB*^{myc} animals shown in Figure 3. NetB is expressed by lamina neurons L3 and medulla neurons (mn), and enriched in the emerging M3 layer at 42 and 55 hr after puparium formation (APF) (arrows, B, C) within the medulla (Me), as well as in the lobula (Lo) neuropils (asterisks). Expression decreases in adults (D). R-cells are labeled with mAb24B10 (red). La, lamina; Me, medulla; Lop, lobula plate; mn, medulla neurons. Scale bars, 20 µm.

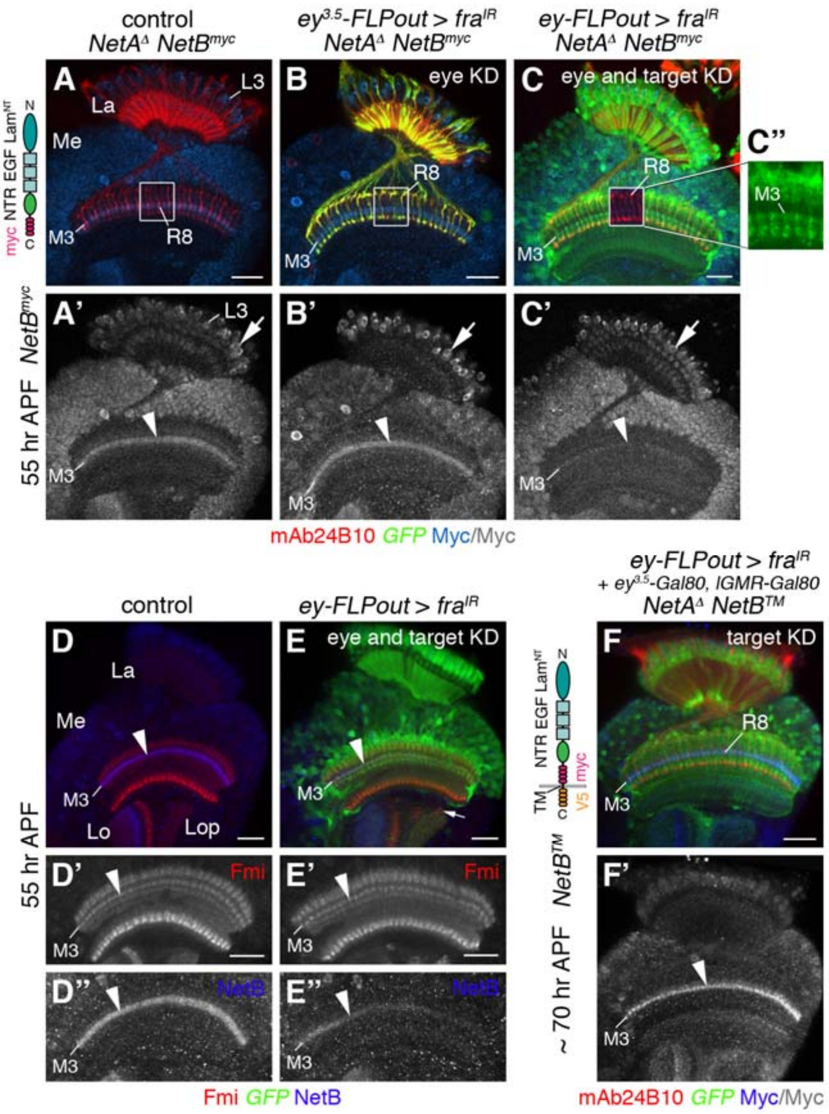

Figure S5, related to Figure 5. Analysis of *NetB^myc^* and *NetB^TM^* Knock-in Transgenes Confirms that Target-derived Fra Captures NetB Protein in the M3 Layer

(A and A’) Control: $\mathrm{NetB}^{\mathrm{Myc}}$ (blue) is enriched in layer M3 (arrowhead, A’), which is innervated by R8 axons (box, A).

(B and B’) *fra* knock-down in the eye using *ey*$^{3.5}$*-FLP, act>>Gal4* FLPout transgenes: $\mathrm{NetB}^{\mathrm{Myc}}$ expression in M3 is unaffected (arrowhead, B’); R8 axons fail to innervate this layer (box, B).

(C-C”) *fra* eye and target area knock-down using *ey-FLP, act>>Gal4* FLPout transgenes: $\mathrm{NetB}^{\mathrm{myc}}$ expression in M3 is strongly decreased, although expression in lamina neuron L3 cell bodies remains high (cf. arrows, A’, B’ and C’). R8 axons show targeting defects (box, C), while GFP expression (C”) indicates that layer M3 has formed. R-cell axons are labeled with mAb24B10 (red).

(D-E”) Control siblings (D-D”, *n*=19) and flies, in which *fra* has been knocked-down in the eye and target area using *ey-FLP, act>>Gal4* FLPout transgenes (E-E”, *n*=14): Co-labeling with Fmi (red) as independent marker and NetB (blue) confirms that layer M3 (arrowhead) has formed normally, while NetB localization is reduced. In controls (D), Fmi is detected within a layer in the lobula (Lo); in flies, in which *fra* has been knocked-down in the eye and target area (E), Fmi expression is also present in the lobula plate (Lop, arrow), suggesting that targeting of higher-order neurons may be affected.

(F and F’) *fra* target area knock-down using *ey-FLP, act>>Gal4* FLPout in conjunction with *ey*$^{3.5}$-*Gal80* and *lGMR-Gal80* transgenes: membrane-tethered NetB ($\mathrm{NetB}^{\mathrm{TM}}$) expression remains high in layer M3 (arrow) of animals, in which *fra* has been knocked-down (*n*=6). La, lamina; Me, medulla. GFP (green) labels the cells, in which the *UAS-fra*^{IR} transgene is expressed.

Schematics show the structure of NetB^{myc} and NetB^{TM} with a laminin N-terminal domain (Lam^{NT}), epidermal growth factor repeats (EGF), Netrin-like domain (NTR), transmembrane domain (TM) and myc and V5 tags.
Scale bars, 20 μm.

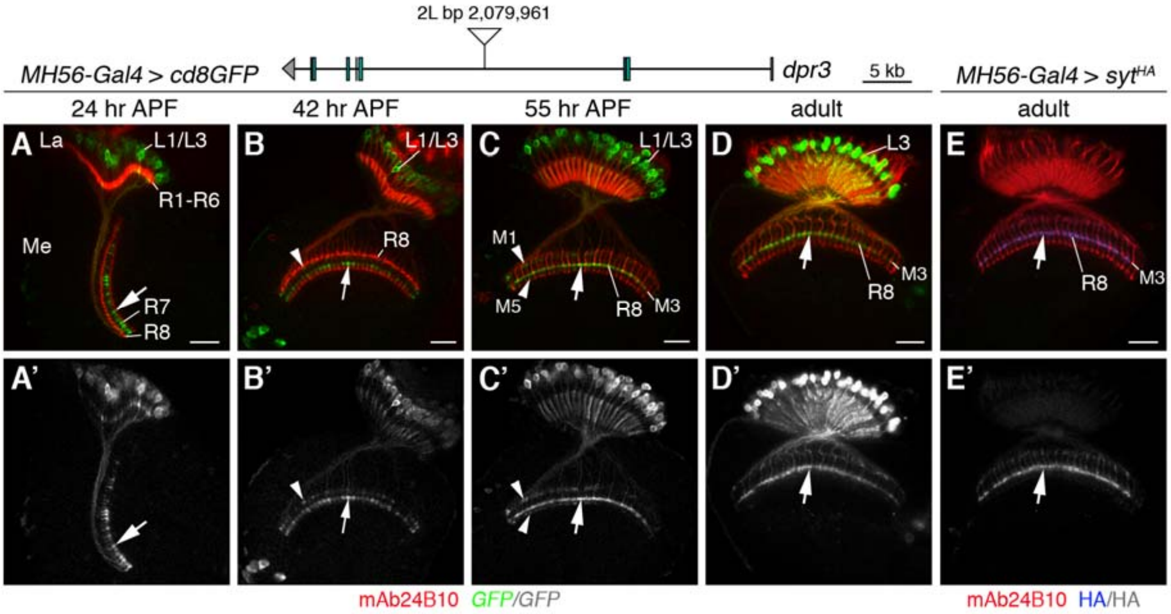

Figure S6, related to Figure 7. Analysis of *MH56-Gal4* Expression Pattern
The P element *MH56-Gal4* is inserted into the *dpr3* locus on the left arm of the second chromosome (2L).
(A-D') At 24, 42 and 55 hr after puparium formation (APF), *MH56-Gal4* drives moderate GFP expression (green) in some lamina neurons L1 extending axonal arbors into layers M1 and M5 (arrowheads). Throughout pupal development and in adults, *MH56-Gal4* drives strong expression in lamina neurons L3 extending axonal arbors into the emerging and final M3 layer (arrows).
(E and E') Expression of *UAS-syt^{HA}* with *MH56-Gal4* confirms that layer M3 represents the output area of this neuron population (arrows), and that this driver is suitable for timely expression of NetB in rescue experiments. R-cell axons are labeled with mAb24B10 (red). La, lamina; Me, medulla.
Scale bars, 20 μm.

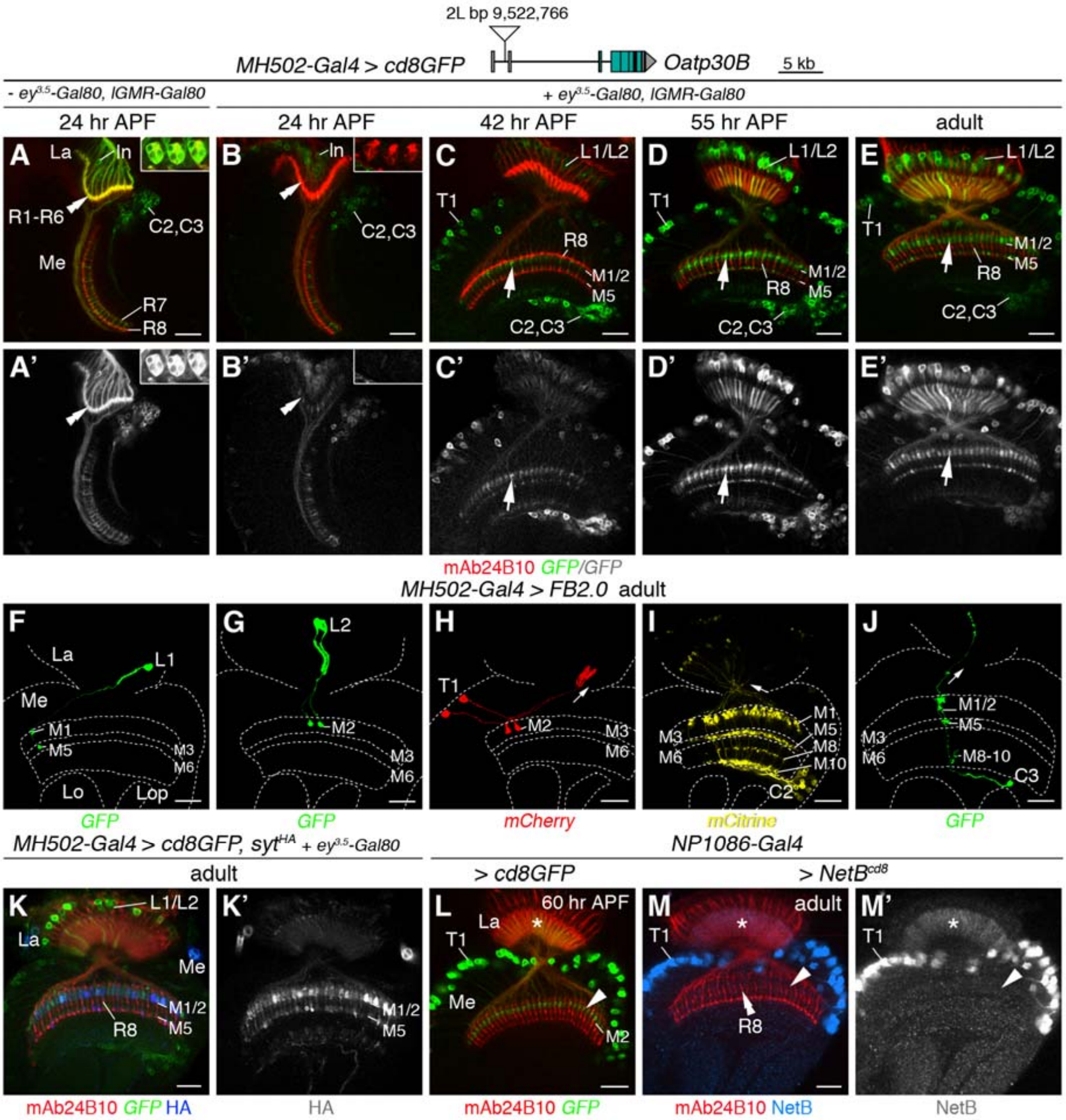

Figure S7, related to Figure 8. Analysis of *MH502-Gal4* Expression Pattern

The P element *MH502-Gal4* is inserted into the *Oatp30B* locus on the left arm of the second chromosome (2L).

(A and A'**)** At 24 hr after puparium formation (APF), *MH502-Gal4* drives GFP expression in R-cells, lamina neurons (ln) and C2/C3 neurons. Strong expression is found in ommatidial clusters in the retina (inset) and in R1-R6 growth cones within the lamina plexus (double-arrowheads).

(B and B') Co-expression of *ey*$^{3.5}$*-Gal80* and *lGMR-Gal80* suppresses *MH502-Gal4* activity in R-cells. GFP expression is strongly reduced in R-cells in the retina (inset) and R1-R6 axons in the lamina (double-arrowheads).

(C-E'**)** At 42 hr, 55 hr APF and in adults, *MH502-Gal4* is active in lamina neurons L1 and L2, and ascending T1 medulla neurons, as well as C2/C3 neurons projecting into the M1/M2 and M5 layers within the distal medulla neuropil.

(F-J**)** Mapping of neurons defined by *MH502-Gal4* expression using *FB2.0* confirms the activity of this driver in lamina neurons L1 and L2, T1 medulla neurons and C2/C3 neurons. Arrows indicate ascending branches into the lamina.

(K and K') Expression of *UAS-syt*^{HA} with *MH502-Gal4* confirms that layers M1/M2 and M5 represent the principal output area of this neuron population within the medulla, and that this driver is suitable for timely expression in ectopic layers.

(K-M') Expression using *NP1086-Gal4*. At 60 hr APF, T1 neurons express GFP within dendrites in M2 (arrowhead) and axons (asterisk) in the lamina (la) (K). NetB^{cd8} is detected within the lamina (asterisk), but not M2 (arrowhead) (L, L'). R8 axons targeted normally to M3 (double-arrowheads, L). R-cells are labeled with mAb24B10 (red). La, lamina; Me, medulla.
Scale bars, 20 μm.

SUPPLEMENTAL TABLES

Table S1, related to Figures 1-8. Full Genotypes of Flies Shown in Main Figure Panels

Figure	Panel	Genotype
Figure 1	C-F'	*caps-Gal4/UAS-cd8GFP*
	G-J'	*wild type*^{OreR}
	K-L'	*ey-FLP/ey*$^{3.5}$*-Gal80; act>y*$^{+}$*>Gal4 UAS-GFP/+; lGMR-Gal80/UAS-Dcr2, UAS-fra*^{IR}
	M, M'	*repo-Gal4/UAS-Dcr2, UAS-fra*^{IR}
Figure 2	A, A'	*Rh6-lacZ/TM2*
	B, B'	*ey*$^{3.5}$*-FLP/+* or *Y; FRT42D Ubi-GFP PCNA*^{775}*/FRT42D fra*^{3} *; Rh6-lacZ/+*
	D-E"'	*elav-Gal4*^{c155}*, UAS-cd8GFP, hs-FLP*^{1}*/+* or *Y; FRT42D tub-Gal80/FRT42D fra*^{3}*; Rh6-lacZ/+*
	F-G'	control: *ey*$^{3.5}$*-FLP/+* or *Y; FRT42D fra*^{3}*/CyO ; ato-τ-myc/+*
	H, H'	*control: ey*$^{3.5}$*-FLP/+* or *Y; FRT42D fra*^{3}*/CyO ; ato-τ-myc/ato-τ-myc*
	I-J'	*ey*$^{3.5}$*-FLP; FRT42D Ubi-GFP PCNA*^{775}*/FRT42D fra*^{3}*; ato-τ-myc/+*
	K, K'	*ey*$^{3.5}$*-FLP; FRT42D Ubi-GFP PCNA*^{775}*/FRT42D fra*^{3}*; ato-τ-myc/ato-τ-myc*
	O, O'	*Rh6-eGFP; lGMR-Gal4*
	P, P'	*Rh6-eGFP/+; lGMR-Gal4/UAS-fra*
Figure 3	A	*NP4151-Gal4/+* or *Y; UAS-cd8GFP/+*
	B	*NP0831-Gal4/+* or *Y; UAS-cd8GFP/+*
	C	*NP4012-Gal4/+* or *Y; UAS-cd8GFP/+*
	D	*R30D09-Gal4/20xUAS-IVS-mcd8GFP*
	E-G'	*hs-FLP*^{1}*/NP4151-Gal4; FB2.0*^{260b}*/hs-mFLP5*
	H	*NetB*$^{CPTI-000168}$
	I	*NetA*$^{CPTI-002152}$
	J-M'	*NetA*$^{\Delta}$ *NetB*^{myc}*/Y* or *FM7*
Figure 4	A, A'	*NetA*$^{\Delta}$*/Y; Rh6-lacZ/+*
	B, B'	*NetA*$^{\Delta}$*/Y; Rh6-lacZ/+*
	C, C'	*NetAB*$^{\Delta}$*/Y; Rh6-lacZ/+*
	E, E'	*ey*$^{3.5}$*-FLP/+* or *Y; act>>Gal4 UAS-GFP/UAS-NetA*^{IR}*; Rh6-lacZ/UAS-Dcr2, UAS-NetB*^{IR}

	F, F'	*ey-FLP/ey$^{3.5}$-Gal80; act>>Gal4 UAS-GFP/UAS-NetA^{IR}; lGMR-Gal80/UAS-Dcr2, UAS-NetB^{IR}*
Figure 5	A, A'	control: *ey$^{3.5}$-Gal80/+* or *Y; ey-FLP/CyO; +/UAS-Dcr2, UAS-fra^{IR}*
	B, B'	*ey$^{3.5}$-FLP/+* or *Y; act>y^{+}>Gal4 UAS-GFP/+; UAS-Dcr2, UAS-fra^{IR}/+*
	C, C'	*ey-FLP/+* or *Y; act>y^{+}>Gal4 UAS-GFP/+; UAS-Dcr2, UAS-fra^{IR} /+*
	D, D'	*ey$^{3.5}$-Gal80/+* or *Y; ey-FLP/act>y^{+}>Gal4 UAS-GFP; lGMR-Gal80/UAS-Dcr2, UAS-fra^{IR}*
	I, I'	*ey$^{3.5}$-Gal80/+* or *Y; ey-FLP/CyO; lGMR-Gal80/UAS-Dcr2, UAS-fra^{IR}*
	J, J'	*ey$^{3.5}$-Gal80/+* or *Y; ey-FLP/act>y^{+}>Gal4 UAS-GFP; lGMR-Gal80/UAS-Dcr2, UAS-fra^{IR}*
	K, K'	*NP4151-Gal4/UAS-syt^{HA}; UAS-cd8GFP/+*
	L, L'	*NP0831-Gal4/UAS-syt^{HA}; UAS-cd8GFP/+*
Figure 6	A-D	*NetA$^{\Delta}$ NetB^{TM}*/Y
	E, E'	*NetA$^{\Delta}$ NetB^{TM}*/Y*; CyO/+; Rh6-lacZ/+*
	J-L	*pGMR-Gal4/FB1.1^{260b}; hs-mFLP5/+*
Figure 7	A	*MH56-Gal4/CyO; UAS-cd8GFP/TM2*
	B	*MH56-Gal4/CyO; UAS-NetB/TM2*
	C, C'	*MH56-Gal4/CyO; UAS-NetB/Rh6-lacZ*
	D, D'	*NetAB$^{\Delta}$/Y; MH56-Gal4/+; Rh6-lacZ/TM2*
	E, E'	*NetAB$^{\Delta}$/Y; MH56-Gal4/+; Rh6-lacZ/UAS-NetB*
Figure 8	B, B'	*Rh6-lacZ/TM2*
	C	*ap-Gal4/+; UAS-cd8GFP/+*
	D	*ap-Gal4/UAS-NetB^{cd8}*
	E, E'	*ap-Gal4/UAS-NetB^{cd8}; Rh6-lacZ/+*
	G	*ey$^{3.5}$-Gal80/+* or *Y; MH502-Gal4/CyO; UAS-cd8GFP/lGMR-Gal80*
	H	*ey$^{3.5}$-Gal80/+* or *Y; MH502-Gal4/+; UAS-NetB^{cd8}/+*
	I, I'	*ey$^{3.5}$-Gal80/+* or *Y; MH502-Gal4/UAS-NetB^{cd8}; Rh6-lacZ/lGMR-Gal80*

Table S2, related to Figures S1-S7. Full Genotypes of Flies Shown in Supplemental Figure Panels

Figure	Panel	Genotype
Figure S1	A, A’	*ey*$^{3.5}$*-FLP/+* or *Y; CyO/+; UAS-Dcr2, UAS-fra*^{IR}*/+*
	B, B’	*ey*$^{3.5}$*-FLP/+ or Y; act>y*$^{+}$*>Gal4 UAS-GFP/+; UAS-Dcr2, UAS-fra*^{IR}*/+*
Figure S2	A, E, E’, G, G’, I, I’, K, M	*ey*$^{3.5}$*-FLP/+* or *Y; FRT42D Ubi-GFP PCNA*^{775}*/FRT42D*
	B, D, F, F’, H, H’, J, J’, L, N, P, R, T, V	*ey*$^{3.5}$*-FLP/+* or *Y; FRT42D Ubi-GFP PCNA*^{775}*/FRT42D fra*^{3}
	C	*wild type*^{OreR}
	O, Q, S, U	*ey*$^{3.5}$*-FLP/+* or *Y; FRT42D fra*^{3}*/+*
Figure S3	A, A’	*ey*$^{3.5}$*-FLP/+* or *Y; FRT42D Ubi-GFP PCNA*^{775}*/CyO; Rh1-lacZ/+*
	B, B’	*ey*$^{3.5}$*-FLP/+* or *Y; FRT42D Ubi-GFP PCNA*^{775}*/FRT42D fra*^{3}*; Rh1-lacZ/+*
	C, C’	*Rh4-lacZ/+*
	D, D’	*ey*$^{3.5}$*-FLP/+* or *Y; FRT42D Ubi-GFP PCNA*^{775}*/FRT42D fra*^{3}*; Rh4-lacZ/+*
Figure S4	A-D’	*wild type*^{OreR}
Figure S5	A, A’	*NetA*$^{\Delta}$ *NetB*^{myc}*/Y*
	B, B’	*NetA*$^{\Delta}$ *NetB*^{myc}*/ey*$^{3.5}$*-FLP; act>y*$^{+}$*>Gal4 UAS-GFP/+; UAS-Dcr2, UAS-fra*^{IR}*/+*
	C-C”	*NetA*$^{\Delta}$ *NetB*^{myc}*/ey-FLP; act>y*$^{+}$*>Gal4 UAS-GFP/+; UAS-Dcr2, UAS-fra*^{IR}*/+*
	D-D”	*ey-FLP/+* or *Y; CyO/+; UAS-Dcr2, UAS-fra*^{IR}*/+*
	E-E”	*ey-FLP/+* or *Y; act>y*$^{+}$*>Gal4 UAS-GFP/+; UAS-Dcr2, UAS-fra*^{IR}*/+*
	F, F’	*ey*$^{3.5}$*-Gal80/NetA*$^{\Delta}$ *NetB*^{TM}*; ey-FLP/act>y*$^{+}$*>Gal4 UAS-GFP; lGMR-Gal80/UAS-Dcr2, UAS-fra*^{IR}
Figure S6	A-D’	*MH56-Gal4/CyO; UAS-cd8GFP/TM2*
	E, E’	*UAS-syt*^{HA}*/+* or *Y; MH56-Gal4/+; +/TM2*
Figure S7	A, A’	*MH502-Gal4/CyO; UAS-cd8GFP/TM6B*
	B-E’	*ey*$^{3.5}$*-Gal80/+* or *Y; MH502-Gal4/CyO; UAS-cd8GFP/lGMR-Gal80*
	F-J	*hs-FLP*^{1}*/+* or *Y; MH502-Gal4/FB2.0*^{260b}*; hs-mFLP5/+*
	K, K’	*UAS-syt*^{HA}*/ey*$^{3.5}$*-Gal80; MH502-Gal4/+; UAS-cd8GFP/+*
	L	*NP1086-Gal4/+* or *Y; UAS-cd8GFP/+*
	M, M’	*NP1086-Gal4/+* or *Y; UAS-NetB*^{cd8}*/+*

SUPPLEMENTAL EXPERIMENTAL PROCEDURES

Molecular Cloning

Constructs were generated using standard cloning techniques. For *pUAS-fra^{IR}*, a 620 bp fragment targeting the Fra transmembrane domain encoding sequence (nucleotides 2497-3058) was generated by PCR amplification using forward fra^{F} 5’CCGGAATTCGGTACCGCCTGCAGAGGAAGATC and reverse fra^{R} 5’GGAAGATCTCCACAATCGACGAGCTGGA primers that added EcoRI and KpnI or BglII restriction enzyme sites, respectively, to facilitate directional cloning as inverted repeats. As spacer the 2nd intron of *engrailed* (*en*) was amplified with 5’TATAGATCTCCAGCGAGCAGTTGG and 5’TTGGATCCCGCTGTAAGTGGAAG primers. The *fra* sequence was inserted in reverse orientation as KpnI and BglII fragment into *pUAST* (Brand and Perrimon, 1993), the spacer was added as BglII and BamHI fragment using BglII to open the vector, and the second *fra* fragment was inserted using EcoRI and BglII. To generate *pUAS-NetB^{IR}*, a 478 bp fragment targeting a part of the Lam^{NT} domain encoding sequence (nucleotides 331-809) was PCR amplified from genomic DNA and inserted as inverted repeats into *pUAST-R57* using KpnI/RsrII and SfiI/XbaI sites (Pili-Floury et al., 2004). *UAS-NetB^{cd8}* was generated by PCR amplification of full-length *NetB* with added KpnI and NotI sites and a *cd8Cerulean^{V5}* fragment (Hadjieconomou et al., 2011a) with added NotI and XhoI sites; these fragments were sequentially subcloned into the modified *pKC26 UAS* vector (Dietzl et al., 2007; Hadjieconomou et al., 2011a) using respective enzymes. Full-length *NetB* was isolated by PCR amplification from flies containing a *UAS-NetB* transgene (Mitchell et al., 1996). Sequence comparison of genomic DNA with that of *UAS-NetB* transgenic flies and our PCR fragment revealed a A793G amino acid change. This alteration does not detectably alter protein function when over-expressed using the Gal4/*UAS* system, as the *UAS-NetB* construct was able to rescue ((Mitchell et al., 1996); this study). Transgenic flies were generated using standard injection protocols. P element-based plasmids were co-injected into *y w* embryos with a *Δ2-3* transposase-expressing plasmid. The *attB*-site containing construct was inserted into *attP*-site containing loci VIE-260b [2L] and VIE-49b [3R] using the germ line-specific *vas-ϕC31* transgene (*vas-ϕ-C31-zh2A*) for X chromosomal integrase expression ((Bischof et al., 2007); for details, see (Hadjieconomou et al., 2011a)).

Genetics

Fly stocks were maintained in standard medium at 25 ºC except for RNAi experiments, for which progeny were shifted to 29 ºC at 24 hours after egg laying (AEL). The following stocks/crosses were used for:

(i) Fra expression - (1) *wild-type^{OreR}*; (2) *caps-Gal4* (Shinza-Kameda et al., 2006) x *UAS-cd8GFP*;

(ii) *fra ey$^{3.5}$-FLP* (Bazigou et al., 2007) - (1) *ey$^{3.5}$-FLP; FRT42D Ubi-GFP PCNA^{775}/CyO* x (2a) *FRT42D*, (2b) *FRT42D fra^{3}/Gla Bc*, (2c) *FRT42D fra^{3}/Gla Bc*; *Rh6-lacZ/TM6B*, (2d) *FRT42D*

fra^{3}/CyO; *Rh1-lacZ/TM6B*, (2e) *FRT42D fra^{3}/Gla Bc*; *Rh4-lacZ/TM6B*, (2f) *FRT42D fra^{3}/Gla Bc*; *ato-t-myc/TM6B*; (3) *ey$^{3.5}$-FLP; FRT42D Ubi-GFP PCNA^{775}/CyO; ato-τ-myc/TM6B* x *FRT42D fra^{3}/Gla Bc*; *ato-τ-myc/TM6B*; (4) *Rh4-lacZ/+* (*fra^{3}*, (Kolodziej et al., 1996));

(iii) *fra* MARCM (Lee and Luo, 1999) - *elav-Gal4^{c155}, UAS-cd8GFP, hs-FLP^{1}; FRT42D tub-Gal80/CyO* x *FRT42D fra^{3}/Gla Bc*; *Rh6-lacZ/TM6B*;

(iv) *fra* RNAi - (1) *repo-Gal4/TM6B* x *UAS-Dcr2, UAS-fra^{IR}*; (2) *ey-FLP; act>y^{+}>Gal4 UAS-GFP/CyO; lGMR-Gal80/TM6B* x *ey$^{3.5}$-Gal80;; UAS-Dcr2, UAS-fra^{IR}* (*lGMR-Gal80* was kindly provided by C. Desplan; *ey$^{3.5}$-Gal80* (Chotard et al., 2005)); (3) *ey$^{3.5}$-FLP; act>y^{+}>Gal4 UAS-GFP/CyO* x *UAS-Dcr2, UAS-fra^{IR}*; (4) *ey-FLP; act>y^{+}>Gal4 UAS-GFP/CyO* x *UAS-Dcr2, UAS-fra^{IR}*;

(v) *fra* gain-of-function analysis - *Rh6-eGFP/CyO*; *lGMR-Gal4/TM6B* x *UAS-fra* (*UAS-fra*, (Kolodziej et al., 1996));

(vi) Netrin expression - (1a) *NP4151-Gal4*, (1b) *NP0831-Gal4*, (1c) *NP4012-Gal4* (Kyoto DGRC; (Hayashi et al., 2002)) x *UAS-cd8GFP*; (2) *GMR30D09-Gal4* x *20XUAS-IVS-mcd8GFP* (Pfeiffer et al., 2008; Pfeiffer et al., 2010); (3a) *NetB$^{CPTI-000168}$*, (3b) *NetA$^{CPTI-002152}$* (Kyoto DGRC); (4) *NetA$^{\Delta}$ NetB^{myc}/FM6* (Brankatschk and Dickson, 2006); (5) *NP4151-Gal4* x *UAS-syt^{HA}/Y; UAS-cd8GFP/+*; (6) *NP0831-Gal4* x *UAS-syt^{HA}/Y; UAS-cd8GFP/+* (*UAS-syt^{HA}* (Chou et al., 2010));

(vii) Flybow (Hadjieconomou et al., 2011a) - (1) *NP4151-Gal4; hs-mFLP5/CyO* x *hs-FLP^{1}; FB2.0^{260b}*; (2) *pGMR-Gal4; hs-mFLP5/TM6B* x *FB1.1^{260b}*; (3) *MH502-Gal4; hs-mFLP5/TM6b* x *hs-FLP^{1}; FB2.0^{260b}*;

(viii) *fra* RNAi and Netrin expression - (1) *ey$^{3.5}$-FLP/Y; act>>Gal4 UAS-GFP/CyO* x *NetA$^{\Delta}$ NetB^{myc};; UAS-Dcr2, UAS-fra^{IR}*; (2) *ey-FLP; act>y^{+}>Gal4 UAS-GFP/CyO* x *NetA$^{\Delta}$ NetB^{myc};;UAS-Dcr2, UAS-fra^{IR}*; (3) *ey$^{3.5}$-Gal80; ey-FLP/CyO; UAS-Dcr2, UAS-fra^{IR}/TM6B* x *act>y^{+}>Gal4 UAS-GFP/CyO; lGMR-Gal80/TM6B*; (4) *ey$^{3.5}$-FLP; act>>Gal4 UAS-GFP/CyO* x *UAS-Dcr2, UAS-fra^{IR}*; (5) *ey-FLP; act>y^{+}>Gal4 UAS-GFP/CyO* x *UAS-Dcr2,UAS-fra^{IR}*;

(ix) *Netrin* loss-of-function analysis - (1) *NetA$^{\Delta}$/FM6; Rh6-lacZ/TM3;* (2) *NetB$^{\Delta}$/FM6; Rh6-lacZ/TM3*; (3) *NetAB$^{\Delta}$/FM6; Rh6-lacZ/TM3* (*Net* alleles, (Brankatschk and Dickson, 2006));

(x) Netrin RNAi - (1) *ey$^{3.5}$-FLP; act>>Gal4 UAS-GFP/CyO; Rh6-lacZ/TM6B* x *UAS-NetA^{IR}/Gla Bc; UAS-Dcr2, UAS-NetB^{IR}/TM6B* (*UAS-NetA^{IR}*, VDRC; *UAS-NetB^{IR}*, this study); (2) *ey-FLP; act>>Gal4 UAS-GFP/CyO; lGMR-Gal80/TM6B* x *ey$^{3.5}$-Gal80; UAS-NetA^{IR}/Gla Bc;UAS-Dcr2, UAS-NetB^{IR}/TM6B*;

(xi) Membrane-tethered NetB (Brankatschk and Dickson, 2006) - (1) *NetA$^{\Delta}$ NetB^{TM}*; (2) *NetA$^{\Delta}$ NetB^{TM}* x *cn bw/CyO;Rh6-lacZ/TM2*; (3) *ey$^{3.5}$-Gal80/Y; ey-FLP/CyO; UAS-Dcr2, UAS-fra^{IR}/TM6B* x *NetA$^{\Delta}$ NetB^{TM}; act>y^{+}>Gal4 UAS-GFP/CyO; lGMR-Gal80/TM6B*;

(xii) Netrin rescue - (1) *MH56-Gal4* x (2a) *UAS-cd8GFP*, (2b) *UAS-NetB* (Mitchell et al., 1996), (2c) *UAS-syt^{HA}/Y; UAS-cd8GFP/+*; (3) *NetAB$^{\Delta}$/FM6; Rh6-lacZ/TM3* x *MH56-Gal4/Gla Bc; UAS-NetB/TM6B*;

(xiii) ectopic expression of membrane-tethered NetB - (1) *ap-Gal4* x *UAS-cdGFP*; (2) *ap-Gal4* x *UAS-NetB^{cd8}/Gla Bc;* (3) *ap-Gal4* x *UAS-NetB^{cd8}/Gla Bc; Rh6-lacZ/TM6B;* (4) *MH502-Gal4/CyO; UAS-cd8GFP/TM6B*; (5) *ey$^{3.5}$-Gal80; MH502-Gal4/CyO;UAS-cd8GFP/TM6B* x *CyO/Gla Bc; lGMR-Gal80;* (6) *ey$^{3.5}$-Gal80; MH502-Gal4/CyO;UAS-cd8GFP/TM6B* x *UAS-Net^{cd8}/TM6B;* (7) *ey$^{3.5}$-Gal80; MH502-Gal4/Gla Bc; Rh6-lacZ/TM2* x *UAS-Net^{cd8}/Gla Bc; lGMR-Gal80/TM6B;* (8) *ey$^{3.5}$-Gal80; MH502-Gal4/CyO; UAS-cd8GFP/TM6B* x *UAS-syt^{HA}*; (9) *NP1086-Gal4* (Kyoto DGRC; (Hayashi et al., 2002)) x *UAS-cd8GFP*; (10) *NP1086-Gal4* x *UAS-NetB^{cd8}*.

Mill Hill (MH)-Gal4 lines were generated in an enhancer trap Gal4 screen to identify drivers active in restricted cell types in the visual system. Inverse PCR was used to determine P element insertion sites. *MH56-Gal4* represents an enhancer trap insertion into the *dpr3* locus (encoding the Ca^{2+} release activated Ca^{2+} channel modulator 2); *MH502-Gal4* is inserted into *Oatp30B*, encoding the Organic anion transporting polypeptide 30B.

For some experiments, CyO balancer chromosomes were marked with *Kr-Gal4, UAS-GFP*. Siblings within crosses served as experimental controls. Over-expression of the *UAS-syt^{HA}* transgene interfered in some samples with R-cell axon targeting; for this study, we focused our analysis on brains displaying a wild type projection pattern.

For Flybow experiments, 24 hour embryo collections were subjected to two 30 min heat shocks at 48 and 72 hours AEL in a 37 ºC water bath.

Histology and Imaging

For immunolabeling of samples shown in Supplemental Figures, the following primary antibodies were used in addition to those listed in the main Experimental Procedures section: guinea-pig anti-Bsh (1:500; (Schmucker et al., 2000)), mouse anti-Dac (1:25; DSHB; (Mardon et al., 1994)), rabbit anti-cleaved Caspase 3 (1:75; Cell Signaling Technology), rat anti-Elav (1:25; DSHB), mouse anti-Fmi (1:50; DSHB), Cy3 conjugated goat anti-HRP (1:200; Jackson ImmunoResearch Laboratories), rabbit-anti-phospho Histone H3 (1:200, Upstate Biotechnology), rabbit anti-Repo (1:500; (Halter et al., 1995)), and guinea-pig anti-Senseless (1:1000; (Nolo et al., 2000)). For immunofluorescence analyses, the following secondary antibodies were used: goat anti-mouse, rabbit, rat and guinea pig F(ab')$_2$ fragments coupled to FITC (1:200), Cy3/DyLight549 (1:400) or Cy5/DyLight649 (1:200) (Jackson ImmunoResearch Laboratories). For 5-ethynyl-2´-deoxyuridine (EdU) labeling, dissected brains were incubated for 1 hour in 10 μM EdU (Click-iT™ EdU Imaging Kit, Invitrogen) in phosphate buffered saline (PBS), fixed for 1 hour in 2% paraformaldehyde (PFA) in 0.1M L-lysine containing 0.05M phosphate buffer, washed in PBS, followed by detection with Alexa Fluor azide 594. Toluidine blue stained semithin sections of adult eyes were prepared as previously described (Salecker and Boeckh, 1995). Light microscopic images were collected using a Zeiss Axioplan light microscope.

SUPPLEMENTAL REFERENCES

Bazigou, E., Apitz, H., Johansson, J., Loren, C.E., Hirst, E.M., Chen, P.L., Palmer, R.H., and Salecker, I. (2007). Anterograde Jelly belly and Alk receptor tyrosine kinase signaling mediates retinal axon targeting in Drosophila. Cell *128,* 961-975.

Bischof, J., Maeda, R.K., Hediger, M., Karch, F., and Basler, K. (2007). An optimized transgenesis system for Drosophila using germ-line-specific phiC31 integrases. Proc Natl Acad Sci U S A *104,* 3312-3317.

Brand, A.H., and Perrimon, N. (1993). Targeted gene expression as a means of altering cell fates and generating dominant phenotypes. Development *118,* 401-415.

Brankatschk, M., and Dickson, B.J. (2006). Netrins guide Drosophila commissural axons at short range. Nat Neurosci *9,* 188-194.

Chotard, C., Leung, W., and Salecker, I. (2005). glial cells missing and gcm2 cell autonomously regulate both glial and neuronal development in the visual system of Drosophila. Neuron *48,* 237-251.

Chou, Y.H., Spletter, M.L., Yaksi, E., Leong, J.C., Wilson, R.I., and Luo, L. (2010). Diversity and wiring variability of olfactory local interneurons in the Drosophila antennal lobe. Nat Neurosci *13,* 439-449.

Dietzl, G., Chen, D., Schnorrer, F., Su, K.C., Barinova, Y., Fellner, M., Gasser, B., Kinsey, K., Oppel, S., Scheiblauer, S., *et al.* (2007). A genome-wide transgenic RNAi library for conditional gene inactivation in Drosophila. Nature *448,* 151-156.

Hadjieconomou, D., Rotkopf, S., Alexandre, C., Bell, D.M., Dickson, B.J., and Salecker, I. (2011a). Flybow: genetic multicolor cell labeling for neural circuit analysis in Drosophila melanogaster. Nat Methods *8,* 260-266.

Halter, D.A., Urban, J., Rickert, C., Ner, S.S., Ito, K., Travers, A.A., and Technau, G.M. (1995). The homeobox gene repo is required for the differentiation and maintenance of glia function in the embryonic nervous system of Drosophila melanogaster. Development *121,* 317-332.

Hayashi, S., Ito, K., Sado, Y., Taniguchi, M., Akimoto, A., Takeuchi, H., Aigaki, T., Matsuzaki, F., Nakagoshi, H., Tanimura, T., *et al.* (2002). GETDB, a database compiling expression patterns and molecular locations of a collection of Gal4 enhancer traps. Genesis *34,* 58-61.

Kolodziej, P.A., Timpe, L.C., Mitchell, K.J., Fried, S.R., Goodman, C.S., Jan, L.Y., and Jan, Y.N. (1996). frazzled encodes a Drosophila member of the DCC immunoglobulin subfamily and is required for CNS and motor axon guidance. Cell *87,* 197-204.

Lee, T., and Luo, L. (1999). Mosaic analysis with a repressible cell marker for studies of gene function in neuronal morphogenesis. Neuron *22,* 451-461.

Mardon, G., Solomon, N.M., and Rubin, G.M. (1994). dachshund encodes a nuclear protein required for normal eye and leg development in Drosophila. Development *120,* 3473-3486.

Mitchell, K.J., Doyle, J.L., Serafini, T., Kennedy, T.E., Tessier-Lavigne, M., Goodman, C.S., and Dickson, B.J. (1996). Genetic analysis of Netrin genes in Drosophila: Netrins guide CNS commissural axons and peripheral motor axons. Neuron *17,* 203-215.

Nolo, R., Abbott, L.A., and Bellen, H.J. (2000). Senseless, a Zn finger transcription factor, is necessary and sufficient for sensory organ development in Drosophila. Cell *102,* 349-362.

Pfeiffer, B.D., Jenett, A., Hammonds, A.S., Ngo, T.T., Misra, S., Murphy, C., Scully, A., Carlson, J.W., Wan, K.H., Laverty, T.R., *et al.* (2008). Tools for neuroanatomy and neurogenetics in Drosophila. Proc Natl Acad Sci U S A *105,* 9715-9720.

Pfeiffer, B.D., Ngo, T.T., Hibbard, K.L., Murphy, C., Jenett, A., Truman, J.W., and Rubin, G.M. (2010). Refinement of tools for targeted gene expression in Drosophila. Genetics *186,* 735-755.

Pili-Floury, S., Leulier, F., Takahashi, K., Saigo, K., Samain, E., Ueda, R., and Lemaitre, B. (2004). In vivo RNA interference analysis reveals an unexpected role for GNBP1 in the defense against Gram-positive bacterial infection in Drosophila adults. J Biol Chem *279*, 12848-12853.

Salecker, I., and Boeckh, J. (1995). Embryonic development of the antennal lobes of a hemimetabolous insect, the cockroach Periplaneta americana: light and electron microscopic observations. J Comp Neurol *352*, 33-54.

Schmucker, D., Clemens, J.C., Shu, H., Worby, C.A., Xiao, J., Muda, M., Dixon, J.E., and Zipursky, S.L. (2000). Drosophila Dscam is an axon guidance receptor exhibiting extraordinary molecular diversity. Cell *101*, 671-684.

Shinza-Kameda, M., Takasu, E., Sakurai, K., Hayashi, S., and Nose, A. (2006). Regulation of layer-specific targeting by reciprocal expression of a cell adhesion molecule, capricious. Neuron *49*, 205-213.
